# Analytical choices drive toxicogenomic potency estimates: a systematic evaluation of transcriptomic points of departure

**DOI:** 10.1093/toxsci/kfag115

**Published:** 2026-09-08

**Authors:** Imke B Bruns, Dayna R Schultz, Emmanuel Demuynck, Friedel Dewulf, Ioannis Theologidis, Steven J Kunnen, Lukas S Wijaya, Ilias Frydas, Nafsika Papaioannou, Elisavet Renieri, Thanasis Papageorgiou, Dimosthenis Sarigiannis, Kyriaki Machera, Birgit Mertens, Jana Asselman, Carsten Weiss, Bob van de Water, Giulia Callegaro

**Affiliations:** Division of Drug Discovery and Safety, Leiden Academic Centre for Drug Research, Leiden University, Leiden, 2333 CC, the Netherlands; Environmental Engineering Laboratory, Department of Chemical Engineering, Aristotle University of Thessaloniki, Thessaloniki, 54124, Greece; HERACLES Research Center on the Exposome and Health, Center for Interdisciplinary Research and Innovation, Balkan Center, Bldg. B, Thessaloniki, 57001, Greece; Scientific Direction of Chemical and Physical Health Risks, Sciensano, Brussels, 1050, Belgium; Department of In Vitro Toxicology and Dermato-Cosmetology, Vrije Universiteit Brussel, Brussels, 1090, Belgium; Blue Growth Research Lab, Ghent University, Oostende, 8400, Belgium; Laboratory of Toxicological Control of Pesticides, Scientific Directorate of Pesticides’ Control & Phytopharmacy, Benaki Phytopathological Institute, Athens, 14561, Greece; Division of Drug Discovery and Safety, Leiden Academic Centre for Drug Research, Leiden University, Leiden, 2333 CC, the Netherlands; Division of Drug Discovery and Safety, Leiden Academic Centre for Drug Research, Leiden University, Leiden, 2333 CC, the Netherlands; Environmental Engineering Laboratory, Department of Chemical Engineering, Aristotle University of Thessaloniki, Thessaloniki, 54124, Greece; HERACLES Research Center on the Exposome and Health, Center for Interdisciplinary Research and Innovation, Balkan Center, Bldg. B, Thessaloniki, 57001, Greece; Environmental Engineering Laboratory, Department of Chemical Engineering, Aristotle University of Thessaloniki, Thessaloniki, 54124, Greece; HERACLES Research Center on the Exposome and Health, Center for Interdisciplinary Research and Innovation, Balkan Center, Bldg. B, Thessaloniki, 57001, Greece; National Hellenic Research Foundation, Athens, 11635, Greece; Environmental Engineering Laboratory, Department of Chemical Engineering, Aristotle University of Thessaloniki, Thessaloniki, 54124, Greece; HERACLES Research Center on the Exposome and Health, Center for Interdisciplinary Research and Innovation, Balkan Center, Bldg. B, Thessaloniki, 57001, Greece; Environmental Engineering Laboratory, Department of Chemical Engineering, Aristotle University of Thessaloniki, Thessaloniki, 54124, Greece; HERACLES Research Center on the Exposome and Health, Center for Interdisciplinary Research and Innovation, Balkan Center, Bldg. B, Thessaloniki, 57001, Greece; Environmental Engineering Laboratory, Department of Chemical Engineering, Aristotle University of Thessaloniki, Thessaloniki, 54124, Greece; HERACLES Research Center on the Exposome and Health, Center for Interdisciplinary Research and Innovation, Balkan Center, Bldg. B, Thessaloniki, 57001, Greece; National Hellenic Research Foundation, Athens, 11635, Greece; Laboratory of Toxicological Control of Pesticides, Scientific Directorate of Pesticides’ Control & Phytopharmacy, Benaki Phytopathological Institute, Athens, 14561, Greece; Scientific Direction of Chemical and Physical Health Risks, Sciensano, Brussels, 1050, Belgium; Blue Growth Research Lab, Ghent University, Oostende, 8400, Belgium; Institute of Biological and Chemical Systems—Biological Information Processing, Karlsruhe Institute of Technology, Eggenstein-Leopoldshafen, 76344, Germany; Division of Drug Discovery and Safety, Leiden Academic Centre for Drug Research, Leiden University, Leiden, 2333 CC, the Netherlands; Division of Drug Discovery and Safety, Leiden Academic Centre for Drug Research, Leiden University, Leiden, 2333 CC, the Netherlands

**Keywords:** transcriptomic point of departure, benchmark concentration, workflow variability, in vitro transcriptomics, next generation risk assessment

## Abstract

Omics technologies are increasingly integrated into next-generation risk assessment, yet quantitative toxicogenomics outcomes remain highly dependent on analytical choices, motivating a systematic evaluation of how bioinformatics workflows influence hazard characterization and transcriptomic Points of Departure (tPOD). Here, we applied 5 independent transcriptomics pipelines to a shared dataset of RPTEC/TERT1 kidney cells exposed to cisplatin across multiple concentrations and time points, comparing effects of pre-processing, benchmark concentration modeling, and pathway-based interpretation strategies. Across workflows, substantial variability was observed in gene-level benchmark concentrations (BMCs). This variability was associated with differences in normalization, filtering, and modeling software, although the present design does not isolate the contribution of individual workflow choices. Despite this variability, convergence increased at later time points as transcriptional responses strengthened, with 24 h consistently identified as the most sensitive time point at the gene level. Aggregation of gene-level BMCs into pathway-based metrics reduced variability but did not eliminate it, with pathway definition emerging as a major determinant of tPOD estimates. Notably, distinct pathway resources showed minimal gene overlap, and smaller, biologically coherent gene sets (e.g. co-expression modules and biomarker panels) produced lower and less dispersed BMCs compared with broader pathway annotations. Furthermore, direct modeling of pathway activity scores yielded systematically different tPODs relative to median-based aggregation, with method-dependent conservativeness influenced by pathway coverage and response strength. Overall, our findings demonstrate that both analytical workflow design and pathway selection critically shape toxicogenomic-derived potency estimates, highlighting the need for harmonized, transparent methodologies to enable robust application of transcriptomics in chemical safety assessment and regulatory decision-making.

There is increasing evidence suggesting that integrating omics technologies, particularly transcriptomics, into chemical risk assessment (CRA) frameworks can enhance hazard characterization and support the derivation of points of departure (PODs) (Mezencev and Subramaniam 2019; del Giudice et al. 2023). Transcriptomics can provide mechanistic insight into stressor mode of action when gene expression changes are translated into functional biological knowledge, particularly within tiered testing strategies that include targeted and organotypic assays (Thomas et al. 2019; Rogers et al. 2025). Accordingly, several frameworks have been developed to guide the use of omics data in CRA and to distinguish adverse from adaptive biological responses (Johnson et al. 2022).

A key application of transcriptomics in hazard assessment is the derivation of transcriptomic PODs (tPODs) (NTP 2018). Standard tPOD workflows typically include quality control and normalization, identification of concentration-responsive genes, benchmark concentration (BMC) modeling of individual genes, post-model quality filtering, and summarization of gene-level BMCs into transcriptome wide or pathway-level tPOD (U.S. Environmental Protection Agency 2012; Crizer et al. 2021; Feshuk et al. 2023; Reardon et al. 2023; Costa et al. 2024; O’Brien et al. 2025). Gene-level BMCs can be summarized using distribution-based approaches, such as percentile methods or the lowest consistent response dose, or gene set-based approaches based on the lowest median BMC among responsive pathways.

Despite the clear advantages and progress of these approaches, several sources of variability must be addressed to strengthen the reliability and regulatory acceptance of tPODs (Aurisano et al. 2023; Gant et al. 2023). These include technical and biological variability in transcriptomic data, differences in preprocessing and modeling workflows, the aggregation of gene-level BMCs into pathway- or distribution-based tPODs, and the challenge of distinguishing adaptive from toxicologically relevant responses (Costa et al. 2024). In addition, experimental design choices, particularly exposure duration in in vitro studies, may strongly influence BMC and tPOD derivation, as supported by recent evidence of time-dependent variation in BMC values (Tennekes and Sánchez-Bayo 2013; Aguayo-Orozco et al. 2018; Carpi et al. 2024; Harrill et al. 2024a).

Several of these challenges have already been addressed through standardized reporting frameworks and published guidance for transcriptomic BMC analyses (OECD 2023; Reardon et al. 2023; Harrill et al. 2024b; O’Brien et al. 2025). However, it remains unclear how much variability arises when independently implemented workflows are applied to the same dataset under real-world conditions. Such comparisons are needed to identify where workflows’ outputs diverge, improve transparency in reporting, and prioritize specific analytical choices for future controlled parameter sensitivity studies. Recent studies have evaluated distribution-based versus gene set-based tPOD approaches (Costa et al. 2024), suggesting broadly similar overall estimates. However, uncertainties remain regarding the robustness of these assessments, particularly with respect to gene set composition and variability in individual tPOD estimates (Beebe-Wang et al. 2022). Further research is needed to optimize gene set selection for tPOD derivation, including the use of annotation-based pathways (e.g. KEGG, WikiPathways, Hallmark) (Kanehisa and Goto 2000; Liberzon et al. 2015; Slenter et al. 2018), curated transcriptomic signatures (e.g. GENOMARK, TGx-DDI) (Li et al. 2015; Ates et al. 2018), or data-driven gene co-expression modules (van Kessel et al. 2026).

In this study, we addressed the points above by applying 5 independent transcriptomic analysis workflows, representative of current practice in the field, to derive tPODs. All workflows were applied to the same transcriptomic dataset, generated in human immortalized renal proximal tubule epithelial cells RPTEC/TERT1 following exposure to the clinically relevant nephrotoxicant cisplatin across 9 concentrations and 6 time points. The OECD Omics Reporting Framework (OORF) was used to systematically document technical choices, including normalization and downstream modeling steps (OECD 2023). Using this framework, we examined how variability introduced during data processing and modeling affects gene-level BMCs, gene set-derived median BMCs, and study-level tPODs. We show that differences in data preprocessing and filtering contribute substantially to variability prior to modeling. The largest observed differences occurred between workflows that also differed in BMD modeling software and several upstream/downstream settings. Post-model filtering criteria can partially harmonize gene-level results across the tested workflows. Temporal analysis revealed a time-dependent convergence in transcriptomic responses, with later time points showing increased coherence in derived BMCs and tPODs. We further demonstrate that pathway-level outcomes depend strongly on pathway definition: large, curated pathways showed greater heterogeneity, whereas system-specific WGCNA gene co-regulation modules and biomarker gene sets yielded more consistent results. Finally, comparisons of median-based and pathway score-based modeling highlighted complementary strengths and limitations, underscoring the importance of pathway-level coverage when deriving pathway-based tPODs. Together, these findings provide a case-study survey of methodological trade-offs and identify analysis decisions requiring harmonization before best practice recommendations for tPOD derivation in Next-Generation Risk Assessment (NGRA)-relevant applications can be made.

## Materials and methods

### Experimental design

RPTEC/TERT1 cells were maintained and differentiated in a 1:1 mixture of DMEM (Thermo Fisher Gibco, 11966-025) and Ham’s F-12 (Thermo Fisher Gibco, 21765-029), yielding a final glucose concentration of 5 mM. The medium was supplemented with 2 mM GlutaMAX (Thermo Fisher, 35050038), 10 ng/ml epidermal growth factor (Merck, E9644), 36 ng/ml hydrocortisone (Sigma-Aldrich, H0888), 5 mg/ml insulin, 5 mg/ml transferrin, 5 ng/ml selenium (Sigma-Aldrich, I1884), and 100 U/ml penicillin with 100 mg/ml streptomycin (Thermo Fisher Gibco, 15070-063). Cells were cultured at 37 °C in a humidified atmosphere with 5% CO_2_. For exposures, cells were seeded into 96-well plates at 25,000 cells per well and cultured for 14 d to achieve a mature proximal tubule phenotype, as described by Aschauer et al. (2015). The medium was refreshed 1 d prior to treatment. At *t* = 0, cells were exposed to cisplatin in a single-exposure design at concentrations of 0.1, 0.5, 1, 2.5, 5, 10, 20, 30, and 50 µM for 4, 8, 16, 24, 48 or 72 h. The concentration range was selected based on a preliminary cytotoxicity assessment, which showed substantial loss of viability at concentrations above 50 µM (Fig. S1A), and on the reported clinical *C*_max_ of cisplatin of 0.87 µg/ml, corresponding to approximately 2.9 µM (Thompson and Joy 2024). The lowest concentration, 0.1 µM, was therefore more than 10-fold below the reported *C*_max_, and the first 4 tested concentrations were below or close to this value. The highest tested concentration, 50 µM, corresponded to approximately 18-fold the reported *C*_max_, to capture a broad transcriptional concentration-response range while avoiding concentrations associated with excessive cytotoxicity. Each cisplatin concentration-time point condition was analyzed using 3 replicate samples. Untreated RPTEC/TERT1 cells were included as time-matched controls, with an additional 6 untreated control samples collected at 0 h. This design yielded 204 samples before quality control. Cisplatin was obtained from the Leiden University Medical Center (LUMC) as a 1 mg/ml solution in 0.9% NaCl (9 mg/ml). Following exposure, cells were washed once with DPBS lacking Ca2+ and Mg2+ and lysed in 50 μl of lysis buffer (1:1 DPBS: TempO-Seq lysis buffer, BioSpyder) prepared at 2× and mixed immediately before use. Plates were sealed with aluminum sealing film (Greiner Bio-One, 676090), incubated for 15 min at room temperature, and stored at −80 °C. Prior to shipment, lysates were thawed and transferred to 96-well conical-bottom plates (Thermo Fisher, 249662), resealed with aluminum film, returned to −80 °C, and shipped on dry ice to BioClavis (United Kingdom). Samples were processed using the TempO-Seq platform with the human whole transcriptome v1.2 probe panel. In May 2023, the probe panel was realigned to the reference genome (GRCh38) using a 96% sequence identity threshold. Standard attenuators were applied according to the manufacturer’s protocol.

### General workflow

#### Quality control and normalization

Each partner followed their own specific pipeline based on pre-existing, laboratory-specific protocols, and all analyses adhered to a shared conceptual framework for deriving BMCs from transcriptomics data adapted from O’Brien et al. (2025) (Fig. 1A). First, quality control (QC) was performed on the raw aligned TempO-Seq counts, including filtering of low-quality probes and exclusion of samples with low library size or poor replicate correlation. After QC, gene-level expression values were derived by aggregating probe counts and performing normalization.

**Fig. 1.**
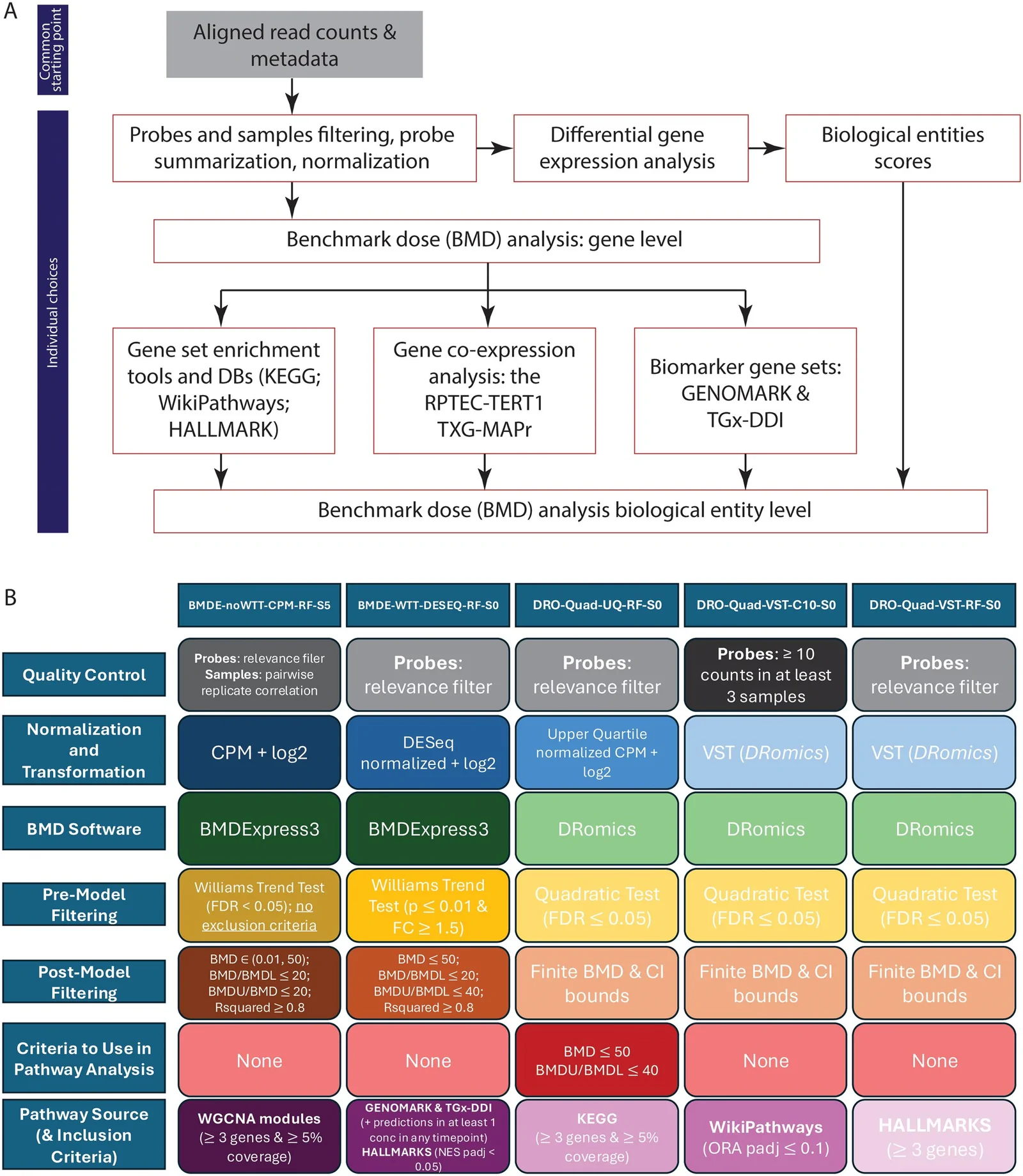
Schematic overview of the analysis workflow. All partners began with the same aligned read counts to minimize variability in preprocessing. From these counts, each partner then carried out their own quality control, normalization, benchmark concentration (BMC) modeling, and pathway interpretation A). Key differences across pipelines included the quality control and normalization steps, the BMD software used, the filtering steps applied, and the pathway sources used B).

#### Determine gene set scores

In some cases, log2 fold changes (log2FC) were calculated using DESeq2 (Love et al. 2014). These log2FC values were also used to generate pathway-level activity scores: either by uploading the data to the RPTEC/TERT1 TXG-MAPr tool to obtain eigengene scores based on a predefined WGCNA network (van Kessel et al. 2026), or by performing gene set enrichment analysis (GSEA) to calculate normalized enrichment scores (NES) (Subramanian et al. 2005).

**Table 1.** Overview of the analytical workflows and naming convention.

Abbreviated workflow identifiers	Full workflow name	BMD software	Prefilter	Normalization	Probe QC	Number of samples removed
BMDE-noWTT	BMDE-noWTT-CPM-RF-S5	BMDExpress3 [*BMDE*]	None [*noWTT*]	Counts per million [*CPM*]	Relevance filter [*RF*]	5 samples removed [*S5*]
BMDE-WTT	BMDE-WTT-DESEQ-RF-S0	BMDExpress3 [*BMDE*]	Williams Trend Test [*WTT*]	DESeq2 [*DESEQ*]	Relevance filter [*RF*]	0 samples removed [*S0*]
DRO-UQ	DRO-Quad-UQ-RF-S0	DRomics [*DRO*]	Quadratic test [*Quad*]	Upper quartile [*UQ*]	Relevance filter [*RF*]	0 samples removed [*S0*]
DRO-VST-C10	DRO-Quad-VST-C10-S0	DRomics [*DRO*]	Quadratic test [*Quad*]	Variance stabilization transformation [*VST*]	Retained probes if they had ≥10 counts in ≥3 samples [*C10*]	0 samples removed [S0]
DRO-VST-RF	DRO-Quad-VST-RF-S0	DRomics [*DRO*]	Quadratic test [*Quad*]	Variance stabilization transformation [*VST*]	Relevance filter [*RF*]	0 samples removed [*S0*]

Abbreviated workflow identifiers are used in the main text to enhance readability, whereas full workflow names are presented in figures and tables to ensure clarity and methodological transparency.

#### Permutation-based empirical null analysis of concentration-responsive gene identification

To assess empirical null-positive behavior at the pre-model concentration-responsive genes (CRGs) identification step, we performed a permutation-based analysis using the workflow-specific prefiltering of CRG-reporting criteria. For each time point, concentration labels were randomly permuted across samples while preserving the original group sizes, and this procedure was repeated 100 times. Each permuted dataset was analyzed up to the CRG-identification step using the same criterion as applied in the corresponding workflow: the Williams Trend Test for BMDExpress3-based workflows, and the quadratic test for DRomics-based workflows. The observed CRG count was compared with the empirical null distribution of the CRG counts obtained from the permuted datasets. The empirical *P*-value was calculated as the proportion of permuted datasets yielding at least as many CRGs as observed in the original dataset, using:


$$\frac{1+\mathrm{number}\;\mathrm{of}\;\mathrm{permutations}\;\geq \mathrm{observed}}{1+\mathrm{number}\;\mathrm{of}\;\mathrm{permutations}}$$

#### Benchmark concentration modeling

Benchmark concentration modeling was applied to normalized gene expression data, yielding gene-level BMCs (Yang et al. 2007; Larras et al. 2018). Due to inherent challenges in summarizing transcriptomic-based BMC data into a single tPOD, multiple strategies were implemented and compared. Specifically, we evaluated 5 gene-level distribution-based approaches for calculating tPODs: the fifth percentile, the 25th lowest-ranked gene, the first mode, the kneedle point, and the lowest consistent response dose (LCRD) (Farmahin et al. 2017). In addition, gene-level BMCs were aggregated at the pathway level, and the median BMC was used to derive a pathway-based tPOD.

To assess the extent to which model-derived estimates were supported by the tested concentration range, gene-level BMCs and derived tPODs were flagged when they fell below the lowest non-zero tested cisplatin concentration of 0.1 μM. Counts and percentages of below-range estimates were summarized by workflow and time point in Table S3.

#### Distribution-based tPODs

The fifth percentile method defines the tPOD as the BMC of the gene nearest to the fifth percentile of the gene-level BMC distribution (Farmahin et al. 2017; Reardon et al. 2021, 2023). The 25th ranked gene approach orders all gene-level BMCs and selects the BMC of the 25th gene (Reardon et al. 2021, 2023; Matteo et al. 2023). Conversely, the first mode identifies the earliest peak in the BMC density curve using a second-derivative inflection-point criterion (Pagé-Larivière et al. 2019). The Kneedle algorithm was applied to identify the point of maximum curvature (“knee-point”) in the ordered BMC distribution (Satopää et al. 2011). Finally, the LCRD is defined as the lowest BMC within the largest group of consecutive BMCs for which the ratio between adjacent values does not exceed a factor of 1.66, forming the consistent group of BMCs (CRGB). Following the adaptation described by Reardon et al. the largest qualifying group is considered rather than the earliest group with subsequent increasing BMCs (Crizer et al. 2021; Reardon et al. 2023).

In addition, to assess the stability of distribution-based tPOD metrics with respect to the set of modeled genes, BMC values were bootstrapped (*n* = 1,000 iterations). In each bootstrap iteration, BMC values were resampled with replacement while preserving the original sample size. For each resampled distribution, tPODs were recalculated, and the resulting distributions were summarized using the median and corresponding 95% CIs.

#### Pathway-based tPODs

To interpret the responses at a higher biological level, genes were grouped into predefined gene sets (e.g. KEGG [Kanehisa and Goto 2000], Hallmark [Liberzon et al. 2015], WikiPathways [Slenter et al. 2018], WGCNA modules [van Kessel et al. 2026], or the TGx-DDI and GENOMARK biomarker panels [Li et al. 2015; Ates et al. 2018]), and pathway-level BMCs were calculated as the median BMC of all concentration-responsive genes in each set. In parallel, BMC modeling was also applied directly to pathway activity scores (eigengene scores or normalized enrichment scores) to enable comparisons between tPODs derived from gene-level aggregation into pathways and those obtained from direct pathway-level modeling (Fig. 1A).

To evaluate the effect of sample size on correlations of Hallmark pathway median BMCs between workflows, we performed a subsampling analysis of gene-level BMCs. For each workflow pair and time point, the number of overlapping pathways contributing to the correlations was determined. Gene-level BMCs were then randomly subsampled 2,000 times, each time selecting the same number of genes as the number of overlapping pathways used in the corresponding pathway-level correlation. Pearson correlations derived from these subsampled gene-level BMCs were subsequently compared with the observed pathway-level median BMC correlations.

#### Details on specific workflows

Details on specific workflows followed by each partner are described in Table S1 and based on selected elements of the OORF (OECD 2023). Key differences are also highlighted in Fig. 1B, and full methodological descriptions are provided in the Supplementary Material. All preprocessing code and partner-specific analysis scripts are accessible in the corresponding GitHub folders and have been archived on Zenodo to provide a permanent, citable record (https://doi.org/10.5281/zenodo.21811562).

To facilitate transparent comparison of analytical approaches, each workflow was assigned a structured identifier reflecting key methodological choices. Workflow names encode the BMD software used, prefilter application, normalization method, probe-level quality control filter, and sample exclusion. For readability, abbreviated identifiers are used throughout the main text, whereas the full workflow names are provided in Table 1.

### Reporting framework for benchmark concentration analysis

To standardize and facilitate the interpretation of concentration-response modeling results across datasets and workflows, we developed a reproducible reporting framework implemented as an R Markdown document. The framework accepts multiple input types, including normalized expression values (i.e. counts per million), log2FC, and pathway-level scores (e.g. BMCs derived from WGCNA eigengene scores or normalized enrichment scores), depending on the analysis performed. For each analysis type, users specify relevant metadata and model output files, after which a series of standardized visualizations and summaries are generated.

The framework is structured into 4 analytical components that summarize and visualize results across different methods. The framework itself does not perform BMC modeling but instead integrates and presents pre-existing analysis outputs in a standardized and interactive format. First, an initial data overview is provided using principal component analysis (PCA) to visualize global transcriptional patterns and assess concentration-dependent separation among samples. Second, gene-level BMCs are summarized to report the number of concentration-responsive genes and interactive accumulation plots. Based on these uploaded results, distribution-based tPODs are also derived, including the fifth percentile BMC, the first mode, the 25th ranked BMC, and the LCRD of the distribution. Third, pathway-level tPODs derived from gene aggregation are summarized using interactive tables, including reported BMC estimates and CIs, with search functionality to enable targeted pathway exploration. In addition, the distributions of gene-level BMCs within the 4 most responsive pathways are visualized in relation to the aggregated pathway BMC and its corresponding CI. Fourth, when pathway score-based BMC modeling results are provided, these outputs are summarized to report concentration-responsive pathways, accumulation plots, and pathway-based tPODs using the same distributional approaches applied at the gene level. If any of the results are not provided, the framework automatically omits that component. All summaries are organized by time point, with separate tabs to facilitate interactive exploration.

The reporting framework was applied independently by each partner, and the resulting HTML reports can be found in the Supplementary Material. In addition, the source code and documentation for the framework is publicly accessible via GitHub (https://github.com/vdWater-Callegaro-lab/PARC-abInitio) and Zenodo (https://doi.org/10.5281/zenodo.21811562).

### Code and data availability

All data analyzed during this study are available in the BioStudies repository under accession number E-MTAB-11374. The code used for data processing, analysis, and figure generation is publicly available on GitHub at https://github.com/vdWater-Callegaro-lab/PARC-abInitio, and a versioned snapshot of the repository has been deposited on Zenodo (https://doi.org/10.5281/zenodo.21811562) to ensure long-term accessibility. Analyses performed by each partner are documented in the corresponding partner-specific folders within the GitHub repository.

## Results

### Gene-level BMC approaches consistently identify 24 h as the strongest responding time point but show workflow-dependent variation in tPOD estimates

#### Data preprocessing before BMC analysis

Each workflow implemented different quality-control steps to the raw count data, including filtering of low-quality probes and, in some cases, exclusion of samples. As a result, the final number of retained genes differed across sites, with 13,040, 12,272, 12,937, 11,531, and 11,531 genes preserved for DRO-UQ, DRO-VST-C10, DRO-VST-RF, BMDE-noWTT, and BMDE-WTT, respectively. As described in the Methods, the experimental design included 204 samples before quality control. BMDE-noWTT excluded 5 samples on quality criteria: one sample (8 h, 0.5 µM) was removed due to a low library size (<500,000 reads), evaluated after filtering low-quality probes, and 4 samples (16 h, 0.1 µM; 48 h 0.5 µM; 72 h 10 µM; 72 h, 20 µM) due to poor replicate correlation. Other workflows retained all samples to maintain a minimum of 3 replicates per concentration-time point condition (Table 2).

**Table 2.** Data dimensions after quality control (QC) for each workflow and normalizations/transformations applied to the data before benchmark concentration modeling.

	Genes after QC (*n*)	Samples after QC (*n*)	Normalization and transformation
BMDE-noWTT-CPM-RF-S5	11,531	199	Log2(CPM)
BMDE-WTT-DESEQ-RF-S0	11,531	204	Log2(CPM)
DRO-Quad-UQ-RF-S0	13,040	204	Upper Quartile
DRO-Quad-VST-C10-S0	12,272	204	Log2(VST) in DRomics
DRO-Quad-VST-RF-S0	12,937	204	Log2(VST) in DRomics

As an initial check of concentration-range adequacy, differential expression was evaluated per concentration and time point using the BMDE-noWTT preprocessing output, prior to the workflow-comparison analyses. This analysis was intended as a descriptive dataset-level check rather than as an additional workflow comparison. Differentially expressed genes (DEGs) showed clearer concentration-dependent increases at later time points, particularly 24, 48, and 72 h, whereas earlier time points showed more variable patterns. These results support the presence of minimally active concentrations for later time points, whereas indicating that early time point responses were less clearly concentration-dependent (Fig. S1B).

#### Number of concentration-responsive genes (CRG) responding to chemical exposure

Selecting significantly responding genes is an important step to limit the number of features entering tPOD analyses and to remove noisy or weak concentration-response patterns. This step was implemented differently across workflows. DRO-UQ, DRO-VST-C10, and DRO-VST-RF applied a quadratic trend test with False Discovery Rate-adjusted *P*-value (FDR < 0.01) in DRomics as a pre-model filter, thereby restricting BMC modeling to genes that showed statistically significant concentration responsiveness. BMDE-WTT and BMDE-noWTT used the Williams Trend Test (WTT; *P*_adj_ < 0.05) in BMDExpress3 to identify concentration-responsive genes; however, BMDE-noWTT did not use WTT as an exclusion criterion before modeling and instead modeled all genes in the dataset and subsequently applied post-model filters to remove poor or noisy BMC fits. Following this strategy, notably, across all time points, only a small fraction of genes (≤2%) did not meet the prefilter criterion but were retained after post-model filtering. Although numerically limited, this subset of genes illustrates that prefiltering decisions can affect the composition of genes considered in downstream tPOD derivation (Fig. S2).

The number of concentration-responsive genes (CRGs) identified during the pre-filter step varied substantially across the different workflows, particularly at the early time points (Table 3). More specifically, at 4 h, DRO-UQ, DRO-VST-C10, and DRO-VST-RF identified 70, 3,618, and 295 CRGs, respectively. These results were obtained after Upper Quartile (UQ) normalization for DRO-UQ and variance-stabilizing transformation (VST) for DRO-VST-C10 and DRO-VST-RF. A key difference between the 2 workflows applying VST normalization lies in the probe-level filtering step. DRO-VST-C10 retained probes with ≥10 reads in at least 3 samples, whereas DRO-VST-RF applied a relevance filter for probe selection. Despite applying the same quadratic trend test within DRomics, substantial differences in the number of CRGs were observed, highlighting the influence of upstream data cleaning and normalization on CRG identification.

**Table 3.** Overview of the criteria applied to define concentration-responsive genes as a pre-filtering step for benchmark concentration (BMC) modeling, together with the number of genes classified as concentration-responsive and the number retained following post-model filtering.

		BMDE-noWTT-CPM-RF-S5	BMDE-WTT-DESEQ-RF-S0	DRO-Quad-UQ-RF-S0	DRO-Quad-VST-C10-S0	DRO-Quad-VST-RF-S0	Mean (CV)
Criteria to identify concentration responsive genes		Williams Trend Test (FDR < 0.05) NOTE: no exclusion criteria	Williams Trend Test (*P* < 0.01 and fold filter ≥ 1.5)	Quadratic Test (FDR < 0.01)	Quadratic Test (FDR < 0.01)	Quadratic Test (FDR < 0.01)	
Concentration-responsive genes (*n*)	4 h	196	649	70	3,618	295	966 (155.2%)
	8 h	3,304	2,833	1,782	5,123	2,347	3,078 (41.4%)
	16 h	3,532	2,807	2,183	3,086	2,866	2,895 (16.9%)
	24 h	8,226	6,477	4,576	5,801	5,298	6,076 (22.9%)
	48 h	7,273	5,593	4,743	6,049	5,581	5,848 (15.8%)
	72 h	8,895	6,401	5,245	6,562	6,028	6,626 (20.6%)
Applied post-model filters		(1) BMD ≥ 0.01 and BMD ≤ 50; (2) BMCU/BMC ≤ 20; (3) BMC/BMCL ≤ 20; (4) Rsquared ≥ 0.8	(1) BMC < 50 μM; (2) BMCU/BMCL ≤ 40; (3) BMC/BMCL ≤ 20; (4) Rsquared ≥ 0.8	(1) Finite BMC point estimates and CI bounds	(1) CI within range of tested doses	(1) Finite BMC point estimates and CI bounds	
Retained genes after post model filters (*n*)	4 h	76	42	66	2,002	268	491 (173.1%)
	8 h	701	695	1,705	2,731	2,004	1,567 (55.9%)
	16 h	1,591	1,552	2,080	2,423	2,357	2,001 (20.6%)
	24 h	3,312	3,149	4,272	4,547	4,471	3,950 (16.9%)
	48 h	4,049	3,562	4,186	4,564	4,483	4,169 (9.6%)
	72 h	5,710	4,061	4,634	5,073	4,982	4,892 (12.4%)

The final column reports the mean number of concentration-responsive genes and the mean number of genes retained after post-model filtering per time point, together with the coefficient of variation.

In addition, the coefficient of variation (CV) was substantially lower at later time points compared with 4 and 8 h, indicating reduced variability in the number of CRGs across workflows at later times. After application of post-model filtering, variability in the number of retained genes was further reduced, with counts converging to a similar range across workflows. More specifically, at 48 h the number of genes retained after post-model filtering ranged from 3,562 for BMDE-WTT to 4,564 for DRO-VST-C10, corresponding to a CV of 9.6%. At 72 h, variability increased slightly, but remained substantially lower compared with early time points, with retained genes ranging from 4,634 for DRO-UQ to 5710 for BMDE-noWTT (CV = 12.4%) (Fig. 2A, Table 3).

**Fig. 2.**
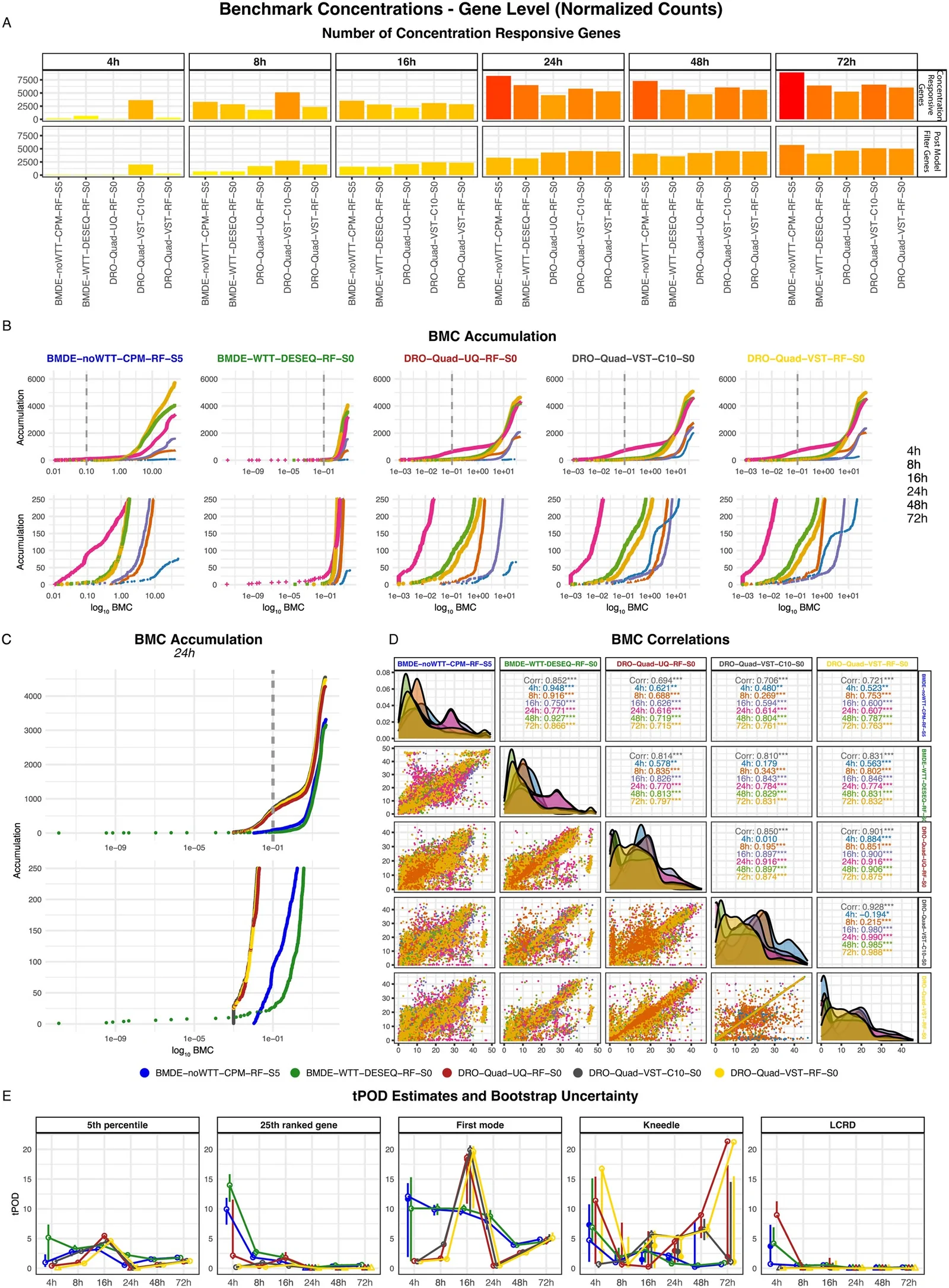
Gene-level BMC distributions and distribution-based tPODs across workflows and time points. Number of dose-responsive genes (CRGs) identified per workflow after application of the prefilter and after subsequent post-model filtering A). Accumulation plots of all modeled genes, colored and grouped by the time point; inset panels show the first 250 genes with the lowest BMC values. Across workflows, the 24 h time point shows the steepest initial accumulation, reflecting a larger number of genes with low BMC values. The vertical dotted line indicates the lowest non-zero tested concentration; BMC estimates to the left of this line fall below the tested concentration range and should be interpreted as below-range model-derived estimates B). Accumulation plots at 24 h comparing workflows, with colors indicating individual workflows C). Gene-level BMC correlation plots between workflows, with colors indicating time points D). Distribution-based tPOD estimates (fifth percentile, 25th ranked gene, first mode, kneedle, and LCRD) with uncertainty assessed by bootstrapping the BMC distributions. Filled circles represent the median bootstrapped tPOD, open circles indicate tPODs derived from the original distributions, and horizontal lines denote the bootstrapped CIs E).

We assessed the overlap of genes passing the pre-filter and post-model filters across workflows. The UpSet analysis of overlapping genes after both pre-filtering and post-model filtering revealed that from 16 h onward, the largest gene set consisted of genes retained across all workflows, suggesting that workflow-specific differences had less influence once transcriptional responses became more pronounced. When considering CRGs after prefiltering, BMDE-noWTT consistently exhibited the highest number of workflow-specific CRGs beyond this shared gene set. This pattern is in line with the differences in how CRGs were defined for reporting and how genes were selected for BMC modeling. DRO-UQ, DRO-VST-C10, and DRO-VST-RF applied a quadratic test with an FDR <0.01 as a pre-model filter, whereas BMDE-WTT used a Williams Trend Test (WTT; *P* < 0.01) combined with an absolute fold-change threshold of ≥1.5 as an exclusion criterion before modeling. In contrast, BMDE-noWTT did not use WTT as a pre-model exclusion criterion; all genes passing QC were modeled, and WTT with FDR < 0.05 was calculated only to report the number of CRGs. Therefore, the larger BMDE-noWTT CRG count reflects the more permissive reporting definition rather than a large pre-filtered modeling set. Conversely, although BMDE-WTT’s statistical threshold is relatively lenient, the additional fold-change requirement appears to exclude a substantial number of genes, leading to fewer genes passing the pre-filter step at later time points (Fig. S3, Table 3).

#### Empirical null analysis of pre-model CRG identification

To evaluate whether workflow-specific CRG-identification criteria also selected genes under a null scenario, we performed a permutation-based empirical null analysis at the pre-model filtering step. Concentration labels were permuted 100 times within each time point while preserving group sizes, and the workflow-specific CRG-identification criteria were reapplied. For DRO-UQ and BMDE-noWTT, the permuted datasets yielded no CRGs in nearly all permutations across timepoints (Fig. S4). The other DRomics-based workflows showed somewhat higher numbers of CRGs under permutation, although this was not specific to a particular time point. In contrast, BMDE-WTT identified at least 1 CRG in all permuted datasets at all time points. This likely reflects the more permissive prefilter used in this workflow, where genes were selected based on a fold-change threshold of >1.5 combined with an unadjusted *P*-value < 0.05, rather than an FDR-adjusted threshold. Consistent with this interpretation, FDR-adjusted *P*-values in this workflow were all > 0.05. Despite differences in null-positive CRG selection, observed CRG counts were generally higher than the permutation-derived null distributions. The only comparison with an empirical *P*-value > 0.05 was BMDE-WTT at 4 h, indicating that the observed number of CRGs at this early time point was not clearly separated from the empirical null distribution (Table S2). This supports cautious interpretation of early time point CRG selection, particularly for workflows using more permissive filtering criteria.

#### Gene-level BMC estimates

Gene-level BMCs were independently derived within each workflow using normalized counts and are presented as accumulation plots per time point (Fig. 2B). Across all workflows, the 24 h exposure showed a steep initial increase in the accumulation curves, indicating that many genes deliver low BMC values (Fig. 2C). This pattern is also reflected in Table 4 and Fig. 2E, where the 24 h time point consistently displayed low distribution-based tPOD estimates (fifth percentile, 25th lowest ranked, first mode, knee-point [Kneedle], and LCRD) across workflows. At later time points, this early response appeared to attenuate, as evidenced by higher overall accumulation levels at 48 and 72 h. In contrast, the earlier time points (4, 8, and 16 h) were characterized by more gradual increases, lower final accumulation levels, and greater variability between workflows (Fig. 2D).

**Table 4.** Distribution-based tPODs.

		BMDE-noWTT-CPM-RF-S5	BMDE-WTT-DESEQ-RF-S0	DRO-Quad-UQ-RF-S0	DRO-Quad-VST-C10-S0	DRO-Quad-VST-RF-S0	Mean
4 h	25th ranked	10 (7.4 to 11.8)	14 (11.2 to 15.8)	2.2 (1.6 to 11.6)	0.2 (0.1 to 0.5)	0.3 (0.1 to 0.8)^b^	5.3
	Fifth percentile	1 (0.2 to 2.4)	5.2 (1.1 to 7.4)	0.4 (0.1 to 0.9)	1.1 (1 to 1.3)	0.1 (0 to 0.2)^a^	1.6
	First mode	12.1 (1.9 to 14.3)	10.1 (1.2 to 15.3)	1.2 (0.9 to 1.5)	1.2 (1 to 1.3)	1.2 (1 to 1.3)	5.2
	Kneedle	4.7 (1.1 to 10.8)	6.9 (4.2 to 15.1)	11.4 (0.7 to 15.4)	0.7 (0.1 to 1)^b^	16.8 (0.6 to 17.3)	8.1
	LCRD	0.7 (0.7 to 7.3)	4.2 (4.2 to 6.9)	9 (9 to 11.3)	0 (0 to 0.5)^a^	0 (0 to 0.7)^a^	2.8
8 h	25th ranked	1.8 (1.4 to 2.8)	2.8 (2 to 3.3)	0.4 (0.2 to 0.7)	0.9 (0.8 to 1.2)	0.2 (0.1 to 0.4)	1.2
	Fifth percentile	2.8 (2.1 to 3.3)	3.3 (2.8 to 3.9)	1 (0.9 to 1.1)	2.9 (2.6 to 3.3)	0.8 (0.8 to 0.9)	2.1
	First mode	9.9 (8.9 to 10.7)	10.1 (9 to 10.9)	1.7 (1.6 to 1.8)	4.1 (3.5 to 4.5)	1.6 (1.5 to 1.7)	5.4
	Kneedle	1 (0.9 to 2.9)	1.9 (0.9 to 3.4)	0.7 (0.1 to 7.7)	1.4 (0.9 to 2.6)	0.6 (0.2 to 5.3)	1.1
	LCRD	0.5 (0.5 to 1)	0.5 (0.5 to 1.4)	0 (0 to 0.2)^a^	0.1 (0.1 to 0.8)	0 (0 to 0.2)^a^	0.2
16 h	25th ranked	1.2 (0.9 to 1.5)	1.9 (1.5 to 2.6)	1.8 (0.5 to 2.7)	0.4 (0.2 to 1)	0.6 (0.3 to 1.5)	1.2
	Fifth percentile	3.2 (2.8 to 3.5)	3.9 (3.3 to 4.4)	5.5 (4.9 to 5.8)	4.6 (3.9 to 5)	4.6 (4 to 5.1)	4.3
	First mode	9.6 (8.9 to 10.2)	10 (9.3 to 10.7)	18.6 (10.9 to 19.3)	19.9 (9.2 to 20.7)	19.7 (10.1 to 20.5)	15.6
	Kneedle	3 (0.6 to 3.4)	3.1 (1 to 4.4)	0.3 (0.1 to 6.2)^b^	5.7 (0 to 6.4)	5.5 (0 to 6.3)^b^	3.5
	LCRD	0.5 (0.5 to 0.6)	0.6 (0.6 to 1)	0.2 (0.2 to 1.8)	0 (0 to 0.9)^a^	0 (0 to 1.3)^a^	0.3
24 h	25th ranked	0 (0 to 0)^a^	0.1 (0 to 0.2)^a^	0 (0 to 0)^a^	0 (0 to 0)^a^	0 (0 to 0)^a^	0.0
	Fifth percentile	0.5 (0.4 to 0.8)	2.2 (2 to 2.6)	0 (0 to 0)^a^	0 (0 to 0)^a^	0 (0 to 0)^a^	0.6
	First mode	8 (6.5 to 9.3)	8.7 (7.3 to 9.8)	0.5 (0.3 to 0.5)	0.4 (0.4 to 0.5)	0.5 (0.4 to 0.5)	3.6
	Kneedle	2.2 (1 to 2.3)	0.8 (0.3 to 2.6)	4.5 (0.9 to 5.1)	5.2 (1.1 to 5.2)	5.2 (1.1 to 5.3)	3.6
	LCRD	0 (0 to 0)^a^	0 (0 to 0.2)^a^	0 (0 to 0)^a^	0 (0 to 0)^a^	0 (0 to 0)^a^	0.0
48 h	25th ranked	0.3 (0.2 to 0.4)	0.5 (0.4 to 0.6)	0 (0 to 0)^a^	0 (0 to 0)^a^	0 (0 to 0)^a^	0.2
	Fifth percentile	1.5 (1.4 to 1.7)	1.5 (1.4 to 1.6)	0.6 (0.5 to 0.7)	0.6 (0.5 to 0.8)	0.6 (0.5 to 0.7)	1.0
	First mode	4 (3.7 to 4.2)	3.9 (3.7 to 4.2)	2.6 (2.4 to 2.8)	2.7 (2.5 to 2.9)	2.6 (2.5 to 2.9)	3.2
	Kneedle	0.2 (0.2 to 7.9)	0.5 (0.4 to 0.7)	9 (5.1 to 9.1)	6.5 (4.7 to 8.3)	6.5 (5.2 to 7.5)	4.5
	LCRD	0 (0 to 0.2)^a^	0 (0 to 0.3)^a^	0 (0 to 0)^a^	0 (0 to 0)^a^	0 (0 to 0)^a^	0.0
72 h	25th ranked	0.4 (0.3 to 0.5)	0.6 (0.5 to 0.8)	0 (0 to 0.1)^a^	0.1 (0 to 0.1)^a^	0 (0 to 0.1)^a^	0.2
	Fifth percentile	1.8 (1.7 to 1.9)	1.8 (1.7 to 1.9)	1.1 (0.9 to 1.3)	1.3 (1.1 to 1.4)	1.2 (1 to 1.4)	1.4
	First mode	4.9 (4.7 to 5.1)	4.4 (4.2 to 4.7)	4.7 (4 to 5.3)	4.9 (4.5 to 5.7)	5.1 (4.6 to 6)	4.8
	Kneedle	0.7 (0.4 to 0.9)	0.9 (0.5 to 1)	21.4 (0.6 to 17.3)	1.1 (0.5 to 14.5)	21.2 (0.5 to 15.5)	9.1
	LCRD	0.2 (0.2 to 0.2)	0.1 (0.1 to 0.3)	0 (0 to 0)^a^	0 (0 to 0)^a^	0 (0 to 0)^a^	0.1

a Indicates that the tPOD estimate is below the lowest tested concentration of 0.1 μM.

b Indicates that the bootstrap resampling interval extends below 0.1 μM. These values should be interpreted cautiously because they fall outside, or are not fully supported by, the observed concentration range. Values below 0.1 μM are retained, but should be considered below-range estimates rather than quantitatively supported potency estimates.

Summary of the distribution-based methods used to derive transcriptomic points of departure (tPODs), including the fifth percentile, 25th ranked gene, the first mode of the distribution, the inflection point (Kneedle) and the lowest consistent response dose (LCRD). Bootstrapped uncertainty is shown in brackets.

Interestingly, the shape of the accumulation plots appears to align with the modeling software used (Fig. 2B). Workflows applying DRomics (DRO-UQ, DRO-VST-C10, and DRO-VST-RF) displayed similar curve shapes across all time points. Similarly, BMDExpress3-based workflows (BMDE-noWTT and BMDE-WTT) showed comparable profiles to each other, with a notable exception that BMDE-noWTT retains a larger number of genes after post-model filters at 72 h, which is also shown in Table 3.

Despite differences in pre-processing and modeling approaches, all workflows consistently identified 24 h as the time point with the steepest increase in the accumulation curves at lower BMC values, indicating that many genes respond at low concentrations. When focusing on 24 h (Fig. 2C), the main separation was observed between the 2 groups of workflows that used different BMD (DRomics versus BMDExpress3). However, when focusing on the 250 CRGs with the lowest BMCs (<3 µM), BMDE-noWTT identified substantially more genes in the lower BMC range than BMDE-WTT, potentially reflecting the stricter prefiltering applied in the BMDE-WTT workflow. More generally, BMCs derived using DRomics tend to be lower, as reflected by the pronounced accumulation near 0 µM.

The BMC correlation matrix (Fig. 2D) further illustrates the degree of similarity between gene-level BMC estimates across workflows. The strongest overall agreement was observed between DRO-VST-C10 and DRO-VST-RF (*r* = 0.928), with correlations exceeding 0.98 from the 16 h time point onward. A notable exception occurred at 4 and 8 h, where DRO-VST-RF consistently estimated low BMC values, whereas DRO-VST-C10 showed greater variability across the concentration range. Correlation between DRO-UQ and DRO-VST-RF was slightly lower (*r* = 0.901) yet remained high (*r* > 0.85) across all time points. In contrast, BMDE-noWTT and BMDE-WTT showed a somewhat lower overall correlation (*r* = 0.852), although their agreement was stronger at earlier time points compared with the DRomics workflows, particularly at 4 h (*r* = 0.948) and 8 h (*r* = 0.916).

Inspection of the correlation plots of gene-level BMCs generated using BMDExpress3 (BMDE-noWTT and BMDE-WTT) revealed a pronounced accumulation of BMC estimates at approximately 28 and 46 µM, visible as horizontal and vertical bands in the figure for 24 and 72 h, respectively. This pattern may reflect a discretization effect in the BMC estimates in BMDExpress3 rather than true biological clustering. Notably, these accumulations are not driven by the same set of genes for both BMDExpress3 workflows, as similar banding patterns are also observed in the correlation plot comparing BMDE-noWTT and BMDE-WTT. Examination of the fitted concentration-response models for genes contributing to the observed banding behavior revealed that the majority of genes with BMCs around 28 µM were described by an exponential 5 (Exp5) model, as shown by the vertical bands in Fig. S5. In addition, genes contributing to the banding observed around 46 µM were predominantly fitted by either an exponential 3 (Exp3) or a power model. These patterns were consistent between BMDE-noWTT and BMDE-WTT, suggesting that the observed banding may be related to BMDExpress3 model-fitting behavior for these specific models rather than workflow-specific gene selection alone (Fig. S5). The pattern may also be influenced by the experimental concentration spacing, particularly the relatively large interval between 30 and 50 µM, which could affect model-derived estimates near the upper end of the tested range. Conversely, this specific banding pattern was not observed in the DRomics-based workflows, which use a continuous model-based approach, suggesting that the artefact may be specific to the BMDExpress3-based BMC estimation procedure.

In addition to these model-dependent patterns within the tested concentration range, a subset of gene-level BMCs fell below the lowest non-zero tested cisplatin concentration of 0.1 µM. We therefore quantified the number and percentage of below-range BMC estimates by workflow and time point (Table S3). Below-range BMCs were more frequent in the DRomics-based workflows, particularly at 24 h, where approximately 15% of retained gene-level BMCs fell below 0.1 µM. In contrast, the proportion of below-range BMCs in the BMDExpress3-based workflows remained 3% or lower across all time points. These estimates were retained as model-derived outputs but should be interpreted cautiously because they fall outside the concentration range directly supported by the experimental design.

Overall, correlations were lowest at early time points (4 to 24 h), but increased markedly at longer exposures, consistently exceeding an *r* of 0.7 at 48 and 72 h. BMC estimates at earlier time points showed greater between-workflow variability, possibly reflecting weaker responses and greater noise. At later time points, when clearer and more consistent responses emerge, the results become more stable and less dependent on the specific analytical approach used.

#### Distribution-based tPODs

Gene-level tPOD estimates were obtained for each time point within each workflow following the BMC reporting framework (see Methods) and applying distribution-based approaches including fifth percentile, 25th ranked gene, first mode, knee-point (Kneedle) and lowest consistent responsive dose (LCRD) of the BMC distribution (see Methods). The LCRD consistently yielded the lowest tPOD values, except for DRO-UQ at 4 h. However, several distribution-based tPODs, particularly some LCRD and some 25th-ranked gene estimates, were below the lowest tested concentration of 0.1 μM and are therefore flagged in Table 4 as below-range estimates, requiring cautious interpretation (Table 4, Fig. S6, Table S3).

To assess the stability of distribution-based tPOD summary metrics with respect to the set of retained gene-level BMCs, the BMC distributions were resampled with replacement using 1,000 bootstrap iterations. The resulting bootstrap intervals therefore reflect resampling variability of the derived distributional metric conditional on the observed set of fitted BMCs. The bootstrap resampling intervals were generally narrower at later time points, suggesting greater stability of the distribution-based summary metrics (Fig. 2E, Table 4). Among the distribution-based metrics, the Kneedle approach exhibited the greatest variability. Notably, the first mode showed a pronounced increase in variability at 16 h in workflows applying DRomics, a pattern that was consistently observed across all DRomics-based workflows. In contrast, the 25th-ranked gene and the LCRD produced highly consistent tPOD estimates at later time points, both in terms of their bootstrapped CIs and their alignment across workflows.

Taken together, these findings indicate that individual gene-level BMC estimates varied substantially across workflows, with differences aligning with upstream normalization choices and the BMD modeling software used. This effect was most pronounced at early time points, when transcriptomic responses were weak or heterogeneous, resulting in substantial variability between workflows. However, when gene-level responses were summarized into distribution-based tPODs, inter-workflow variability was markedly reduced, which was particularly evident from 24 h onwards. Additionally, uncertainty associated with tPOD estimation decreased at later time points, as reflected by narrower bootstrapped CIs, indicating increased stability.

### Reduced correlation of aggregated BMCs at the hallmark pathway level is largely driven by feature count

In addition to distribution-based tPODs, pathway-level aggregation was used to derive tPODs by summarizing gene-level BMCs into median pathway BMC values, allowing assessment of pathway-specific responses and potential insights into the biological mode of action. Applying a common set of Hallmark pathways enabled direct comparison of pathway-level responses across workflows, thereby providing insight into whether aggregating functionally related genes reduced inter-workflow variability trends observed at the gene-level (Liberzon et al. 2015).

The resulting pathway-level accumulation curves (Fig. 3A) indicated that 48 h generally yielded the lowest pathway-level tPOD estimates across workflows, except for DRO-VST-RF, highlighting a divergence from the gene-level analysis. Compared with the gene-level curves, the pathway-level accumulation profiles are less strongly associated with the BMD modeling software used. Interestingly, DRO-VST-RF shows a steep increase in the accumulation curve at 4 h, suggesting that some pathways responded more intensely at this early time point. However, this pattern is likely influenced by the absence of a pre-filter requiring a minimum percentage of pathway coverage or significant over-representation to define biologically active pathways and may be resulting from an overall minimum overlap of 3 genes. Specifically, at 4 h in the DRO-VST-RF workflow, only 3 of the 34 active pathways showed ≥5% gene coverage: p53 (12 genes), MTORC1 (11 genes), and TNFα signaling (15 genes). Additionally, the overrepresentation analysis identified only p53 and TNFα signaling as significant (FDR = 0.024 and FDR = 0.00357, respectively). In contrast, both DRO-UQ and BMDE-noWTT applied an additional requirement of at least 5% pathway coverage and, accordingly, did not identify any active pathways at 4 h.

**Fig. 3.**
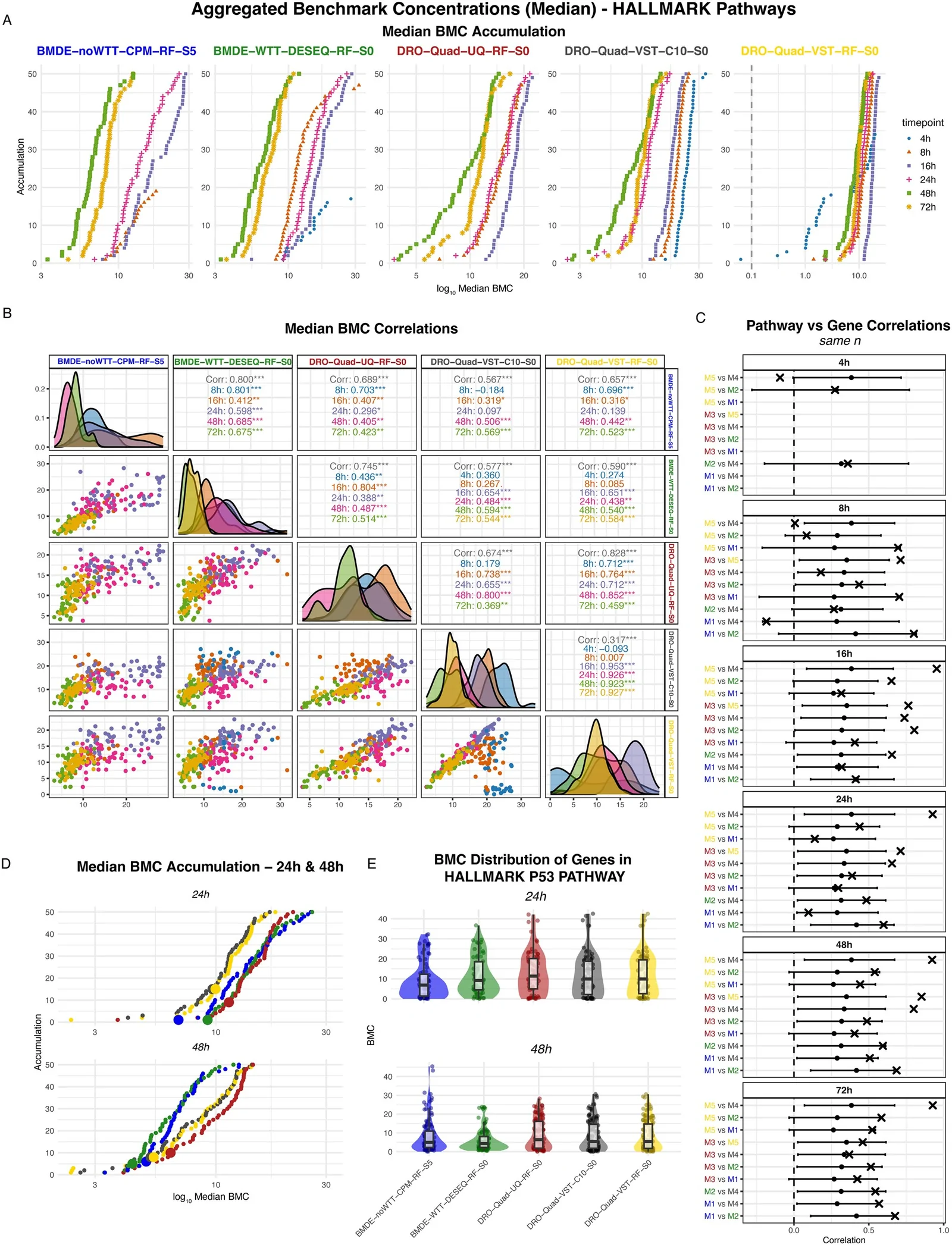
Pathway-level median BMCs derived from Hallmark gene sets across workflows and time points. Accumulation plots of aggregated median BMCs for Hallmark pathways, with colors indicating time points A). Correlation plots of pathway-level median BMCs derived from Hallmark gene sets between workflows, with colors indicating time points. The vertical dotted line indicates the lowest non-zero tested concentration B). Observed pathway correlations versus gene-bootstrap expectations. The x marks the observed pathway correlation between workflows. Points and error bars indicate the mean and 95% CI obtained by sampling the same number of genes as overlapping pathways for each workflow and time point. M1 = BMDE-noWTT-CPM-RF-S4, M2 = BMDE-WTT-DESEQ-RF-S0, M3 = DRO-Quad-UQ-RF-S0, M4 = DRO-Quad-VST-C10-S0, M5 = DRO-Quad-VST-RF-S0 C). Accumulation plots of Hallmark pathways at 24 and 48 h, shown per workflow (colors), highlighting the Hallmark p53 pathway D). Distributions of gene-level BMCs within the Hallmark p53 pathway for each workflow at 24 and 48 h E).

Overall, the strongest agreement across workflows in pathway-level median BMCs was observed at 48 and 72 h. Although pathway-level correlations were generally lower than gene-level correlations, this reduction is partly driven by the smaller number of pathway-level data points. Notably, sampling an equivalent number of genes resulted in poorer correlations (Fig. 3C), indicating that pathway-level aggregation provides comparatively higher cross-workflow concordance at matched dimensionality. Furthermore, increased variability was observed among workflows using DRomics, particularly at early time points. This was most evident between DRO-VST-C10 and DRO-VST-RF at 4 h (*r* = −0.149) and 8 h (*r* = −0.036), whereas correlations improved substantially at later time points (*r* ≥ 0.77 from 16 h onwards). The highest overall correlation was observed between DRO-UQ and DRO-VST-RF (overall *r* = 0.828). Their correlations exceeded 0.7 at all time points except 72 h, contrasting with the gene-level analysis, where correlations consistently increased at later time points.

BMDE-noWTT and BMDE-WTT also showed high overall agreement (overall *r* = 0.800), despite lower gene-level concordance compared with DRomics-based workflows. Nevertheless, variability between BMDE-noWTT and BMDE-WTT was most pronounced at 16 h (*r* = 0.412) and 24 h (*r* = 0.598), mirroring the time points at which reduced correlations were observed in the gene-level analysis.

From a pathway-oriented toxicological perspective, Fig. 3D and E illustrate that workflow-specific analytical choices were associated with different tPOD estimates, even within the well-established cisplatin-responsive Hallmark p53 pathway (Table 5) (Rabik and Dolan 2007; Basu and Krishnamurthy 2010; Thompson and Joy 2024). At 24 h, the p53 pathway ranked lowest by median BMC in both BMDE-noWTT and BMDE-WTT. In contrast, the DRomics-based workflows yielded lower median BMCs for other pathways, including but not limited to E2F targets, G2M checkpoint, Hypoxia, and TNFα signaling (Table S4).

**Table 5.** Aggregated median BMCs for p53-related pathways across workflows.

	BMDE-noWTT-CPM-RF-S5	BMDE-WTT-DESEQ-RF-S0		DRO-Quad-UQ-RF-S0	DRO-Quad-VST-C10-S0	DRO-Quad-VST-RF-S0
	**P53 Pathway (Hallmark)**					
4 h	N/A	15.3		N/A	21.3	15.8
8 h	11.2	10.8		12.7	15.7	12.6
16 h	9.6	11.2		12.3	13.0	12.8
24 h	6.9	9.2		11.4	9.7	10.0
48 h	5.0	4.4		6.4	5.2	5.4
72 h	7.0	5.9		6.9	8.2	7.9

	**Pathway definition of choice**					
	**hRPTECTERT1_35 (WGCNA)**	**GENOMARK84**	**TGxDDI64**	**p53 signaling pathway (KEGG)**	**p53 transcriptional gene network (WikiPathways)**	**P53 Pathway (Hallmark)**

4 h	N/A	N/A	N/A	N/A	23.0	15.8
8 h	14.6	12.3	10.0	7.0	13.5	12.6
16 h	6.7	9.5	9.5	13.1	14.0	12.8
24 h	2.5	5.7	7.9	8.3	9.0	10.0
48 h	2.6	2.9	2.7	2.1	2.6	5.4
72 h	2.5	4.1	3.0	3.9	6.4	7.9

The upper section presents median BMC values for the Hallmark p53 pathway across all workflows and time points. The lower section summarizes median BMCs for workflow-specific p53 pathway definitions, including the KEGG p53 signaling pathway, the WikiPathways p53 transcriptional gene network, the Hallmark p53 pathway, the hRPTECTERT1_35 WGCNA module, and the GENOMARK and TGx-DDI biomarker gene sets, as applied by DRO-UQ, DRO-VST-C10, DRO-VST-RF, BMDE-noWTT, and BMDE-WTT, respectively.

Despite these differences in ranking, aggregated median BMC values for the Hallmark p53 pathway were comparable across workflows, ranging from 6.9 for BMDE-noWTT to 11.4 for DRO-UQ at 24 h, and converged further at 48 h (4.4 for BMDE-WTT to 6.4 for DRO-UQ). Examination of the underlying gene-level BMC distributions within the Hallmark p53 pathway (Fig. 3D) revealed that at 24 h, BMDE-noWTT and BMDE-WTT exhibited slightly reduced dispersion of gene-level BMCs, contributing to lower median pathway BMCs. At 48 h, a stronger accumulation of genes at lower BMC values was observed across all workflows, resulting in generally lower and more comparable median BMC estimates.

Together, these findings show that although pathway-level aggregation may yield more stable cross-workflow agreement than gene-level comparisons at equivalent sample size, it does not fully remove the variability present at the gene level. Rather, a substantial proportion of this variability persists when gene-level BMCs are aggregated into median pathway BMC values.

### Variable gene content across p53 pathway definitions leads to divergent tPOD estimates, with smaller pathways showing reduced gene-level dispersion

To more accurately reflect a real-world application scenario, we conducted a follow-up comparison in which each workflow selected a representative, intentionally diverse biological pathway resource for analysis. DRO-UQ used KEGG pathways (Kanehisa and Goto 2000), DRO-VST-RF selected MSigDB Hallmark gene sets (Liberzon et al. 2015), DRO-VST-C10 utilized WikiPathways (Slenter et al. 2018), BMDE-noWTT applied their in-house RPTEC/TERT1 WGCNA modules (van Kessel et al. 2026), and BMDE-WTT employed the GENOMARK and TGx-DDI biomarker panels (Li et al. 2015; Ates et al. 2018), which have been previously identified as transcriptomic biomarkers of genotoxicity. This allowed for an assessment of how diverse database architectures may influence tPOD outcomes.

Given that cisplatin primarily exerts its effects through p53 signaling (Rabik and Dolan 2007; Basu and Krishnamurthy 2010; Thompson and Joy 2024), we focused specifically on p53-related signaling networks within each resource. To explore how pathway definitions may contribute to differences in functional interpretation, we examined the overlap in gene content among the p53 pathways selected by each workflow. Specifically, the KEGG p53 signaling pathway (hsa04115; *n* = 52 genes), the WikiPathways p53 transcriptional gene network (*n* = 96 genes), and the Hallmark p53 pathway (*n* = 200 genes) were included. In addition, the RPTEC/TERT1 WGCNA module hRPTECTERT1_35 (*n* = 33 genes), annotated as a p53 response module, was evaluated alongside the GENOMARK (*n* = 84 genes) and TGx-DDI (*n* = 64 genes) gene sets. Strikingly, not a single gene was shared across all pathway sets. Furthermore, only 1 gene (DDB2) was present in 5 of the selected gene sets, whereas most genes were unique to a single pathway database (Fig. 4A). This limited overlap indicates that gene sets intended to represent similar biological processes can differ substantially in their underlying gene composition, which may influence pathway-level BMC estimates and downstream biological interpretation.

**Fig. 4.**
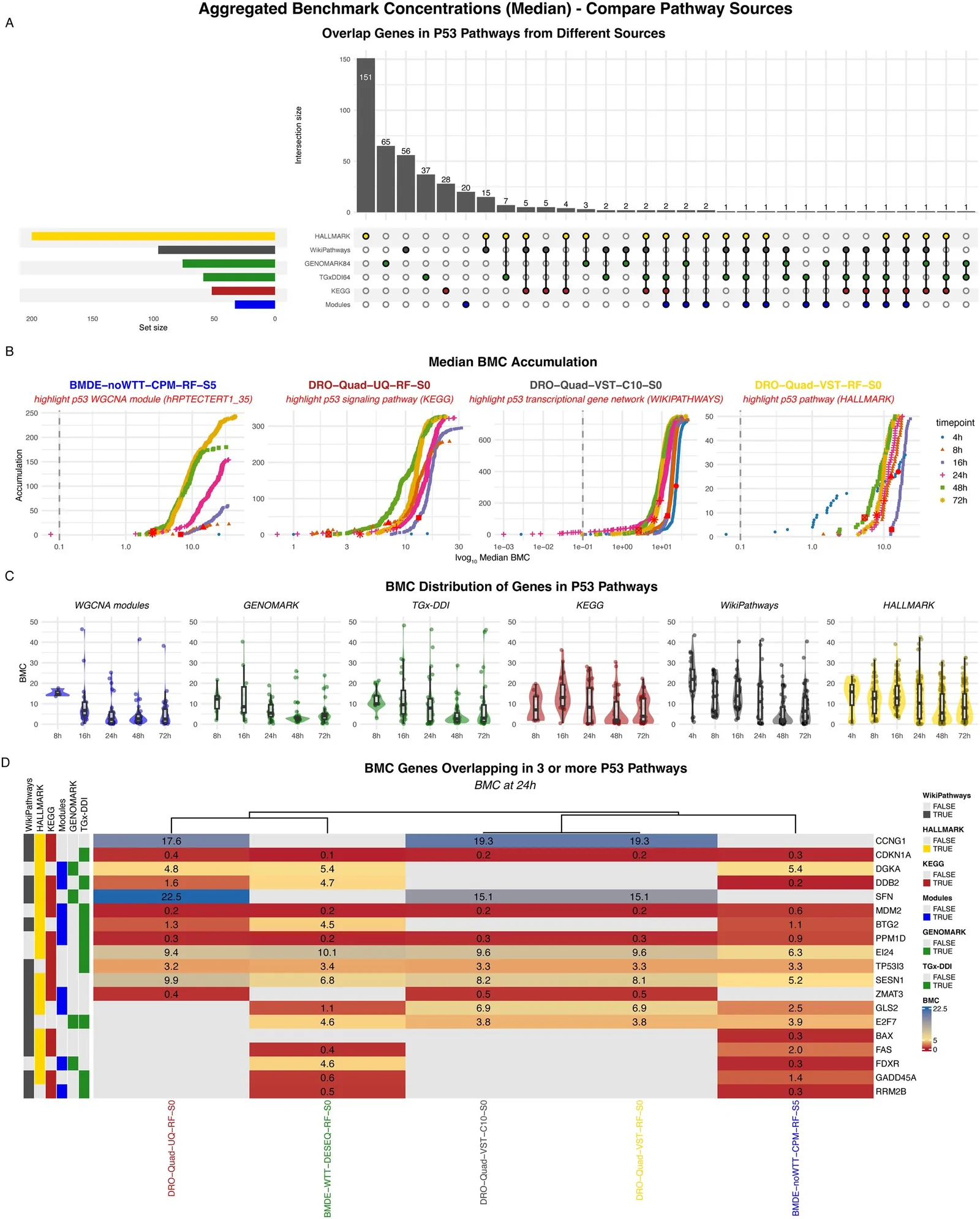
Comparison of aggregated benchmark concentrations (BMCs) derived from different p53 pathway definitions. UpSet plot showing gene overlap among p53-related pathway definitions, including the Hallmark p53 pathway (yellow), WikiPathways p53 signaling pathway (gray), genotoxicity biomarker gene sets GENOMARK and TGxDDI (green), the KEGG p53 signaling pathway (red), and the hRPTECTERT1_35 WGCNA module annotated as a p53 response (blue) A). Accumulation plots of median BMCs aggregated for each pathway source, with p53-related pathways highlighted in red and colors indicating time points. The vertical dotted line indicates the lowest tested non-zero concentration B). Distributions of gene-level BMCs within each p53 pathway source C). Heatmap of genes overlapping in 3 or more p53 pathway definitions. Colors indicate BMC values (red = low BMC, blue = high BMC), and annotations on the left denote the pathway sources in which each gene is included D).

To assess how the choice of pathway source influences BMC determination, we compared the median BMCs derived from these different biological entities. The median BMC for the p53-related pathway was calculated for each gene set and highlighted in the accumulation plot (Fig. 4B). Additionally, we examined the distribution of individual gene-level BMCs within each pathway and observed that larger pathways, which are typically found in resources like KEGG, WikiPathways, and Hallmark, tended to yield higher median BMCs and exhibited greater dispersion in gene-level BMC estimates (Fig. 4C, Table 5). In contrast, more compact and functionally cohesive sets such as WGCNA modules and biomarker gene sets showed narrower BMC distributions and generally lower median BMC values. Notably, this pattern was observed despite the absence of strict pre-model filtering. As shown previously, a subset of genes that did not meet the prefilter criterion were nonetheless retained after post-model filtering (Fig. S2). Among the genes mapped to the p53-related pathways, several retained genes showed consistently low BMC estimates, which is compatible with a biologically plausible cisplatin-associated DNA-damage response.

Lastly, we evaluated whether genes that are more consistently associated with the p53 network exhibit lower BMC values by examining genes shared by at least 3 out of 6 p53-related pathway definitions. This analysis revealed that genes with consistently low BMCs across all workflows (PPM1D, MDM2, and CDKN1A; BMC < 1 µM for all) were identified as p53-related by at least one of the more specific pathway definitions, such as the RPTEC/TERT1 WGCNA modules or the biomarker gene sets. In contrast, genes with higher BMC values (SFN and CCNG1; >15 µM) were predominantly annotated as p53-related in the broader pathway resources (Hallmark, KEGG, and WikiPathways). Generally, most of the core p53 genes show comparable BMC values across workflows. For example, BMC estimates for TP53I3 ranged from 3.2 to 3.4 µM, and CDKN1A showed BMCs between 0.1 and 0.4 µM across all workflows (Fig. 4D). Similar levels of agreement were observed for most of the remaining core p53 genes, indicating a high degree of consistency in gene-level BMCs within this core gene set.

In summary, these findings show that pathway definition influences gene set-based tPOD estimates. Larger, broadly defined pathways (e.g. KEGG, Hallmark, and WikiPathways) exhibited greater variability in gene-level BMCs, often resulting in higher median BMC values compared with more compact system-specific (WGCNA) or curated biomarker (GENOMARK or TGx-DDI) sets. In contrast, core p53 genes shared across multiple pathway definitions showed consistent BMC values across workflows and pathways, highlighting the importance of careful pathway curation when deriving pathway-based tPODs.

### Direct modeling of pathway activity scores yields distinct tPOD estimates compared with median gene-level BMC aggregation

Building on the pathway-level BMC analysis, we next applied BMC modeling directly to pathway activity scores. This score-based approach was used to capture coordinated pathway responses rather than the concentration-responsive behavior of individual genes within pathways. Two complementary types of scores were evaluated: normalized enrichment scores (NES) obtained from fast GSEA of Hallmark pathways, and eigengene scores derived from projecting log2FC values onto the existing RPTEC/TERT1 TXG-MAPr network. This approach allowed us to evaluate how pathway-level modeling compares with median aggregation of gene-level BMCs.

Figure 5A illustrates the accumulation of the pathway-level responses, with the p53 signaling pathway highlighted as an example. Based on eigengene scores (EGs) and the BMDE-noWTT workflow, the p53 pathway was not concentration-responsive at 4 and 8 h but became responsive from 16 h onward. Interestingly, this pattern aligns with the median-based BMC values obtained for pathways derived from the RPTEC/TERT1 TXG-MAPr (as described previously). In the median-based analysis, the 8 h time point also met the inclusion threshold of at least 3 genes passing all model filters and a minimum of 5% pathway coverage. However, the p53 pathway at 8 h contained exactly 3 qualifying genes, suggesting that this signal may be an artifact of lenient filtering.

**Fig. 5.**
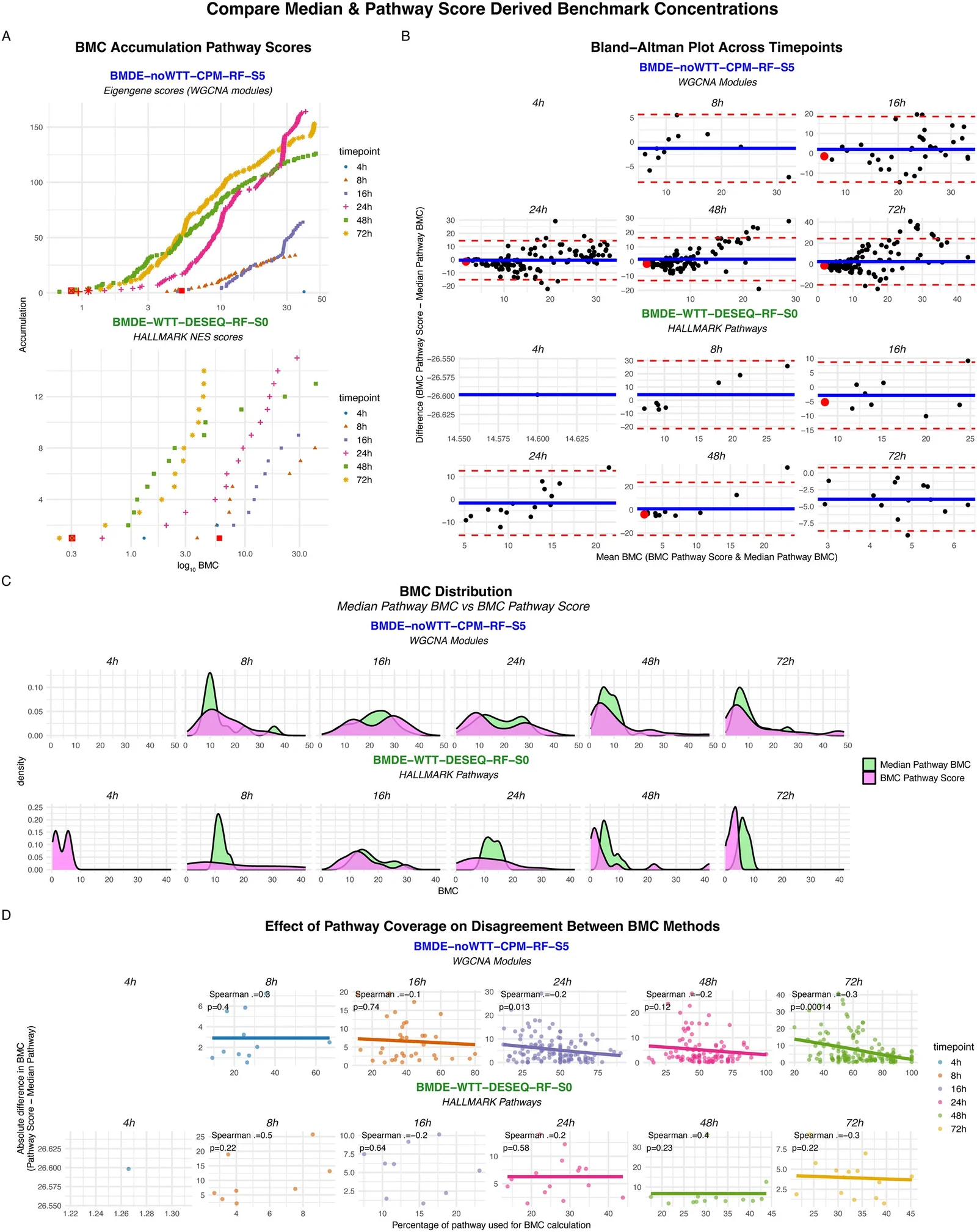
Benchmark dose modeling based on pathway scores compared with pathway-level aggregation. Accumulation plots of benchmark concentrations (BMCs) derived from direct modeling of pathway scores. BMDE-noWTT modelled WGCNA eigengene (EG) scores, whereas BMDE-WTT modeled normalized enrichment scores (NES) from Hallmark pathways. Colors indicate time points, and the p53-related pathways (hRPTECTERT1_35 or Hallmark p53) are highlighted in red A). Bland-Altman plot comparing BMCs derived from direct modeling of pathway scores with aggregated median BMCs for the corresponding pathways or modules. The blue line indicates systematic bias between methods, and the red dotted lines represent the limits of agreement. The p53-related pathway or module is highlighted in red B). Distributions of BMCs obtained from pathway score modeling (pink) and aggregated median pathway BMCs (green) C). Relationship between pathway coverage and disagreement between BMC derivation methods. The *y*-axis shows the absolute difference in BMC values (pathway score-based minus aggregated median), and the *x*-axis shows the percentage of pathway genes contributing to the aggregated median BMC D).

To compare pathway definitions based on co-expression modules with those derived from curated gene sets, we next evaluated normalized enrichment scores (NES) for the Hallmark pathways. The BMDE-WTT workflow yielded an accepted model fit for the Hallmark p53 pathway at 16 and 48 h, but not at other time points. However, the score-based BMC values for the Hallmark p53 pathway were highly similar to those obtained for the score-based hRPTECTERT1_35 WGCNA module, with values of 6.0 and 5.3 at 16 h, and 0.3 and 0.8 at 48 h, respectively. This similarity indicates that, despite differences in gene composition (10 genes overlap), score-based modeling captures a consistent p53-related response, resulting in comparable BMC estimates when the pathway response can be reliably modeled. Importantly, the inability to model pathway scores at certain time points does not necessarily indicate absence of pathway activation but likely reflects limitations of modeling pathway scores.

#### Pathway score-based modeling yields more conservative benchmark concentrations for strong responses

Comparison of score-based BMCs with median-based BMCs revealed a systematic pattern. At the lower end of the BMC distribution, score-based pathway modeling produced lower BMC estimates compared with median-based approaches. At the upper end of the distribution, however, the median-based estimates were relatively lower, indicating that the direction of conservativeness depended on the magnitude of the pathway BMC (Fig. 5B and C). This behavior was observed consistently in both EG- and NES-based analyses.

Consistent with this pattern, when focusing on the p53 signaling pathway, score-based BMCs were lower than the median-based BMCs (Table 6). This suggests that pathways showing strong activation, with many CRGs in the pathway, pathway-level score-based modeling may yield lower BMC estimates by capturing the integrated pathway response rather than individual gene effects. At the same time, these findings underscore the absence of a universally “most conservative” approach, as the relative stringency of each method depends on the strength and coherence of the underlying pathway response.

**Table 6.** Aggregated median BMCs and pathway score-based BMCs for WGCNA eigengene scores (EGs) and Hallmark normalized enrichment scores (NES) across time points.

	WGCNA hRPTECTERT1_35 (BMDE-noWTT-CPM-RF-S5)		Hallmark P53 Pathway (BMDE-WTT-DESEQ-RF-S0)	
	Median BMC	Pathway score (EGs) based	Median BMC	Pathway score (NES) based
4 h	N/A	N/A	15.3	N/A
8 h	14.6	N/A	10.8	N/A
16 h	6.7	5.3	11.2	5.9
24 h	2.5	0.9	9.2	N/A
48 h	2.6	0.8	4.4	0.3
72 h	2.5	1.1	5.9	N/A

N/A denotes pathways excluded after post-model filtering or not classified as responsive (e.g. fewer than 3 responsive genes for BMDE-noWTT and BMDE-WTT, or <5% gene coverage for BMDE-noWTT).

As differences between score-based and median-based BMC estimates may reflect how comprehensively a pathway is represented, we next evaluated whether the extent of pathway coverage influenced the differences observed between median BMCs and pathway score-based BMCs, under the hypothesis that greater gene coverage would reduce discrepancies between the 2 approaches. For the WGCNA modules, pathway coverage was significantly associated with the absolute BMC difference at 24 h (*P* = 0.013) and 72 h (*P* = 0.00014), indicating improved agreement between the 2 approaches when a larger fraction of the module was represented in the median BMC calculation. In contrast, at lower coverage levels (e.g. around 30% at 48 h), substantial variability between the 2 BMC estimates was observed (Fig. 5D).

Taken together, pathway score-based modeling and median aggregation yielded systematically different BMC estimates, with their relative conservativeness depending on gene coverage. Overall, for strongly responding pathways such as the p53 pathways following cisplatin exposure, score-based modeling consistently yielded lower BMC estimates for both NES and EGs. In contrast, median gene-level aggregation was more conservative for pathways with weaker responses.

## Discussion

In this study, we evaluated how methodological choices across independently implemented workflows influence transcriptomic BMC and tPOD derivation. By comparing normalization, modeling, and pathway-level aggregation strategies, we show that methodological variability can substantially affect BMC estimates. The largest differences in BMC and tPOD estimates were observed between workflows that used different BMD modeling software. However, because these workflows also differed in normalization, filtering, BMR selection, model sets, and post-model filtering criteria, the present study cannot attribute the observed differences to any single workflow parameter. Instead, these results should be interpreted as an exploratory comparison of complete, real-world analytical workflows. Despite these differences, workflow outputs became more concordant when transcriptomic responses were stronger, particularly at later time points. Gene- and pathway-level analyses also provided complementary views of the concentration-response data, with pathway-level estimates additionally influenced by pathway definition, gene set size, and gene coverage. Together, these findings highlight major sources of variability in transcriptomic concentration-response analysis and underscore the need for transparent reporting, harmonized evaluation criteria, and targeted follow-up studies to assess individual workflow choices in NGRA-relevant applications.

Variation in data normalization and filtering introduced substantial differences in the data prior to BMC modeling, which is in line with previous findings (Haber et al. 2018; Mezencev and Auerbach 2020). This effect is reflected in discrepancies between workflows using the same modeling software but applying different preprocessing strategies. This underscores the importance of streamlined and standardized preprocessing workflows (e.g. R-ODAF) within NGRA frameworks to reduce variability from quality control and normalization steps and to support more consistent interpretation of transcriptomic data for regulatory decision-making (Verheijen et al. 2022).

In addition, prefiltering can lead to large differences in the set of genes retained for modeling, especially at early time points when transcriptomic responses are noisy (Webster et al. 2015; Farmahin et al. 2017; Nöth et al. 2025). Interestingly, when prefiltering was not used as an exclusion criterion, gene retention after post-model filtering was highly comparable across workflows, suggesting that post-model criteria alone can help harmonize gene selection. This was particularly evident at later time points, where many genes that did not meet the prefilter criterion were nevertheless retained after post-model criteria, including goodness-of-fit thresholds such as *R*^2^ ≥ 0.8. This indicates that these genes produced concentration-response patterns that were modellable under the applied post-model criteria, although their potential false-positive contribution cannot be determined from these filters alone. Such discrepancies are consistent with the behavior of trend-based statistical tests, such as the Williams Trend Test (WTT), which are designed to detect evidence of monotonic concentration-related changes but do not evaluate the shape or quality of fitted concentration-response models (Bretz 2006), as opposed to a quadratic trend test in DRomics, which evaluates linear and non-linear (curvature) trends across concentration levels (Delignette-Muller et al. 2023). As a result, genes showing relatively shallow concentration-response trends may be excluded by prefiltering, underscoring the importance of carefully balancing statistical significance thresholds with model-based performance criteria when defining CRGs.

Consistent with the time-dependent stabilization of transcriptomic responses, the time point associated with the strongest response differed by analysis level. At the gene-level, 24 h yielded the lowest distribution-based tPOD estimates, whereas pathway-level aggregation of gene BMCs generally yielded the lowest pathway-level estimates at 48 h, which is in line with previous findings (Chauhan et al. 2016). This shift may reflect progressive coordination of individual gene responses into coherent biological pathways over time, resulting in more integrated and stable pathway-level perturbations. From an NGRA perspective, these findings highlight that the choice of sampling time point can influence tPOD interpretation and should be aligned with the intended level of biological interpretation. In this RPTEC/TERT1 cisplatin dataset, 24 h yielded the lowest gene-level distribution-based tPOD estimates, whereas 48 h generally yielded the lowest pathway-level estimates. Although these specific time points should not be generalized across compounds or cell systems, the observed sequence is biologically plausible: individual gene-level responses may become detectable before coordinated pathway-level perturbations are captured. Therefore, evaluating multiple exposure durations may be important when transcriptomic data are used to support both early molecular-response detection and pathway-level biological interpretation (Kinaret et al. 2020).

Cross-workflow correlations of pathway-level BMCs (Hallmark) were generally weaker than those observed at the gene level. This reduction likely reflects both aggregation of heterogeneous gene responses and the smaller number of pathway-level features, as subsampling gene-level BMCs to a comparable number of data points similarly reduced correlations. In contrast, pathway-level BMCs for the p53 pathway at 48 h converged across workflows within a comparable range, consistent with its well-established role in the mode of action of cisplatin (Rabik and Dolan 2007; Basu and Krishnamurthy 2010; Thompson and Joy 2024). Thus, although pathway aggregation may reduce overall inter-workflow concordance, it can yield consistent estimates when pathway responses are strong.

To reflect a more real-world scenario, we evaluated how pathway definition influences pathway-level BMCs. Comparison of p53-related pathways derived from different sources revealed substantial heterogeneity in gene composition (Bao et al. 2022; Beebe-Wang et al. 2022). No single gene was shared across all 5 p53 pathway definitions considered, and only 1 gene (DDB2) overlapped across 5 of the 6 pathways (Bao et al. 2022). These findings highlight that pathway-level results are highly dependent on pathway annotation choices, even when representing the same biological process (Mubeen et al. 2020).

To explore whether more focused gene sets could mitigate variability in pathway-level BMCs, we examined the dispersion of gene-level BMCs within different pathway definitions. As expected, larger curated pathways exhibited greater dispersion in individual gene BMCs, reflecting their broad biological scope. This heterogeneity increases the likelihood that only a subset of pathway genes is actively driving the pathway-level response. In contrast, gene-level BMCs within WGCNA-derived modules and curated biomarker gene sets were less dispersed, indicating more coherent transcriptional responses. This aligns with the design of WGCNA modules, which are data-driven and system-specific, as well as the curated biomarker gene sets such as GENOMARK and TGx-DDI that represent defined stress and DNA damage responses (Li et al. 2015; Ates et al. 2018; Costa et al. 2024). Interestingly, gene-level BMC estimates from a core set of p53-associated genes, defined as genes present in at least 3 of the evaluated p53 pathway definitions, were highly consistent across workflows. This suggests that, despite variability in broader pathway definitions, a well-curated core gene set consistently captured the central transcriptomic response to cisplatin and yielded comparable BMC estimates across analytical workflows. Overall, pathway-level analyses are informative when pathway composition, gene-level coverage, and aggregation criteria are carefully considered. Aggregating BMCs across large and heterogeneous gene sets, such as Hallmark, KEGG, or WikiPathways, can yield unstable pathway-level estimates when gene coverage is low or when only a small subset of genes drives the summary metric. Applying minimum gene-coverage criteria and evaluating complementary evidence such as overrepresentation analysis may improve the interpretability of pathway-level tPODs and reduce overinterpretation of weakly supported pathway signals. Refining aggregation strategies and focusing on biologically coherent core gene sets may further enhance the reliability of pathway-based tPODs for NGRA (Khatri et al. 2012).

In addition to median aggregation of gene-level BMCs, alternative strategies exist that derive BMCs directly from pathway-level activity scores (Harrill et al. 2024b). We therefore compared pathway-score derived BMCs (using Hallmark normalized enrichment scores [NES] and eigengene scores (EGs) derived from WGCNA modules) with median-based BMCs from the same pathway sources. Overall, pathway score-based modeling yielded lower BMC estimates at the lower end of the BMC distribution (Harrill et al. 2024a), whereas at higher BMC levels the relative conservativeness shifted toward median-based aggregation. It is important to note that failure to derive BMCs from NES- or EG-based modeling does not imply a lack of pathway activation but may instead reflect challenges in fitting a robust concentration-response model. Enrichment-based scores aggregate large gene sets, which can dilute strong signals from a subset of responsive genes (Subramanian et al. 2005). In contrast, median aggregation is highly influenced by gene selection and pathway coverage, as it relies on the subset of genes passing post-model filters. This likely underlies the lower BMC estimates observed at higher BMC ranges, particularly when pathway coverage is limited. For NGRA applications, score-based modeling may therefore better capture coordinated pathway-wide responses, whereas median aggregation remains more dependent on the subset of retained genes mapped to each pathway and should therefore be interpreted alongside coverage metrics.

Several limitations of this study should be acknowledged. First, the analyses were conducted using a single reference compound, cisplatin, in one cell model, RPTEC/TERT1, and under one experimental design. Although cisplatin provided a useful benchmark because of its well-characterized mode of action and strong transcriptomic response (Thompson and Joy 2024), the conclusions should be interpreted as specific to this compound, cell type, exposure design, and concentration range. In addition, untreated rather than vehicle-treated cells were used as controls. Although cisplatin was prepared in 0.9% saline, an approximately isotonic vehicle that is not expected to strongly perturb cells at low final dilution, the absence of matched saline-treated controls means that the potential vehicle-, dilution-, or handling-related effects cannot be formally excluded. Third, the concentration range constrained quantitative tPOD interpretation. The lowest tested concentration, 0.1 μM, was not demonstrated to be transcriptionally inactive at all time points, and several BMCs or tPODs were estimated below this concentration. These estimates may therefore be below-range values rather than well-supported quantitative potency estimates. Finally, this study was designed as an exploratory comparison of complete, independently implemented workflows, reflecting how tPOD analyses may differ across institutions in a real-world setting, rather than as a factorial assessment of individual workflow parameters. Because workflows differed simultaneously in preprocessing, normalization, filtering, BMR selection, model sets, BMD software, post-model filtering, and pathway definitions, the observed differences cannot be attributed to a single analytical choice. Instead, these findings highlight how non-harmonized analytical decisions can lead to variable gene- and pathway-level outputs, underscoring the need for more transparent and systematically evaluated workflows. Differences in BMC estimates or tPOD derivations should therefore be interpreted descriptively, rather than as evidence of relative sensitivity, specificity, or best-practice performance. The shared BMC reporting framework provides a step towards transparent comparison, whereas follow-up studies using replicated experiments, empirical null analyses, and controlled workflow perturbations will be important to assess experimental variability, workflow-specific false-positive behavior, and the contribution of individual analytical choices, respectively (Cao et al. 2012; Replicability 2019). Broader evaluation across additional chemicals and cell types will also be needed before generalizing these findings to toxicogenomic analyses for NGRA more broadly.

In conclusion, using a shared RPTEC/TERT1 cisplatin exposure dataset, we show that methodological variability across independently implemented workflows was associated with differences in derived tPODs, underscoring the need for transparent reporting, workflow harmonization, and controlled parameter-sensitivity studies. Although omics technologies offer unprecedented resolutions to characterize molecular perturbations, our comparison of 5 independent analysis workflows shows that even when using the same experimental dataset, differences in preprocessing choices, BMD modeling tools, pathway selection, and tPOD derivation methods can lead to markedly different downstream results, emphasizing the need for higher-level methodological harmonization.

## Supplementary Material

kfag115_Supplementary_Data
