# Supplementary material for: Analytical choices drive toxicogenomic potency estimates: a systematic evaluation of transcriptomic points of departure
Source: Toxicol Sci. 2026 Sep 8;209(9):kfag115. doi: 10.1093/toxsci/kfag115 (PMC13623630; doi:10.1093/toxsci/kfag115)
Supplement: Supplementary file 1 [file kfag115_supplementary_data.docx]

**Analytical Choices Drive Toxicogenomic Potency Estimates: A Systematic Evaluation of Transcriptomic Points of Departure**

Imke B. Bruns^1^, Dayna R. Schultz^2,3^, Emmanuel Demuynck^4,5^, Friedel Dewulf^6^, Ioannis Theologidis^7^, Steven J. Kunnen^1^, Lukas S. Wijaya^1^, Ilias Frydas^2,3^, Nafsika Papaioannou^2,3,8^, Elisavet Renieri^2,3^, Thanasis Papageorgiou^2,3^, Dimosthenis Sarigiannis^2,3,8^, Kyriaki Machera^7^, Birgit Mertens^4^, Jana Asselman^6^, Carsten Weiss^9^, Bob van de Water^1^, Giulia Callegaro^1,*^

**Affiliations:**

^1^Division of Drug Discovery and Safety, Leiden Academic Centre for Drug Research, Leiden University, Leiden, the Netherlands.

^2^Aristotle University of Thessaloniki, Thessaloniki, Greece.

^3^HERACLES Research Center on Health and the Exposome, Center for Interdisciplinary Research and Innovation, Aristotle University of Thessaloniki, Greece

^4^Scientific Direction of Chemical and Physical Health Risks, Sciensano, Brussels, Belgium

^5^Department of In Vitro Toxicology and Dermato-Cosmetology, Vrije Universiteit Brussel, Brussels, Belgium.

^6^Blue Growth Research Lab, Ghent University, Ghent, Belgium.

^7^Laboratory of Toxicological Control of Pesticides, Scientific Directorate of Pesticides’ Control & Phytopharmacy, Benaki Phytopathological Institute, Kifissia, Greece.

^8^National Hellenic Research Foundation, Athens, Greece.

^9^Institute of Biological and Chemical Systems - Biological Information Processing, Karlsruhe Institute of Technology, Karlsruhe, Germany.

# **Supplementary Material**

## **Supplemental Methods**

### BMDE-noWTT-CPM-RF-S5 workflow

#### TempO-Seq Data Preprocessing

TempO-Seq analysis was performed using a whole transcriptome probe panel, and raw read counts were provided by BioClavis. All subsequent analyses were performed in R (version 4.5.0). To ensure data quality, several preprocessing steps were applied. First, probe-to-gene mapping was performed using the Human Whole Transcriptome 1.2 manifest (realigned in 2023). Next, a relevance filter adapted from the R-ODAF pipeline was applied, resulting in the removal of 8477 probes (Verheijen et al., 2022). Probe counts mapping to the same gene were aggregated, yielding 11531 unique genes for downstream analysis.

Samples with a total library size below 500,000 reads were excluded, leading to the removal of one sample. To assess replicate quality, pairwise correlations were calculated among replicates within each treatment group. For each group, a correlation threshold was defined as the maximum pairwise correlation minus three times the interquartile range (3*IQR). Replicates with a maximum pairwise correlation below this threshold were excluded. This step resulted in the removal of three additional samples, for a total of four samples excluded from further analysis. Finally, raw counts were normalized to counts per million (CPM), followed by a log2 transformation after adding a pseudo count of 1 to avoid infinite values, enabling comparison across samples.

#### Differential Expression Analysis

Differential expression analysis was conducted using DESeq2 (version 1.48.2) (Love et al., 2014). Departing from the standard DESeq2 workflow, which typically estimates a single log2 fold change (log2FC) per treatment condition by aggregating replicates, we adapted the analysis to calculate log2FC values for each replicate individually. For this, each treatment replicate was compared against the pooled control replicates (n=6) at the corresponding timepoint. This approach was necessary to generate replicate-level log2FC values required as input for downstream analysis in BMDExpress3.

#### TXG-MAPr module projection

To assess biological responses at the pathway level, log2FC values were uploaded in the TXG-MAPr platform (Callegaro et al., 2021). This tool enables the projection of new gene expression data onto a precomputed Weighted Gene Co-Expression Network Analysis (WGCNA) map (Langfelder & Horvath, 2008). In our case, the map was previously generated in-house using a large RPTEC-TERT1 dataset, which included transcriptomic profiles from exposures to over 50 compounds across multiple concentrations and two timepoints (van Kessel et al., 2025). The resulting network consists of 291 gene co-expression modules representing diverse biological processes. By projecting our log2FC values onto this reference map, we obtained eigengene scores summarized expression profiles for each module in every sample, facilitating interpretation of the underlying biology in a structured pathway-level context.

#### Concentration-Response Modeling

##### Gene Level Analysis

Benchmark concentration modeling was conducted using BMDExpress3 (version 3.20) on normalized expression values (log2CPM), and eigengene scores as described above (Yang et al., 2007). No Williams Trend Test (WTT)-based prefiltering was applied before Benchmark Concentration (BMC) modeling, as we aimed to retain all quality control (QC)-passing genes for modeling without exclusion based on preliminary statistical tests. However, the WTT was still performed for reporting purposes to identify concentration-responsive (CRGs), using an FDR-adjusted p-value threshold of < 0.05, and no fold-change cutoff. Genes not passing these filters were not excluded from subsequent BMC modeling. The BMC analysis utilized the suite of EPA BMDS models implemented in BMDExpress3, including Hill, Power, Linear, Polynomial (2^nd^ and 3^rd^ order), and Exponential (3^rd^ and 5^th^ order) models. Benchmark response (BMR) thresholds were defined using the standard deviation method with a BMR factor of 1.021 (5%).

Post-modeling, genes with benchmark concentrations (BMCs) below 10% of the lowest tested concentration (0.01 µM) or above the highest tested concentration were excluded. To ensure reliable concentration-response estimates, genes were further filtered based on model uncertainty: those with a BMCL/BMC or BMC/BMCU ratio >= 20 were removed. Finally, genes with a model fit R^2^ < 0.8 were excluded to ensure adequate model performance.

##### Pathway Level Analysis

All concentration-responsive genes that passed the previously described quality filters were assigned to gene sets. For functional annotation and cross-workflow comparison, we used the MSigDB HALLMARK gene sets (2023) (Liberzon et al., 2015). Additionally, WGCNA-derived RPTEC/TERT1 modules were also treated as gene sets to obtain a BMC value per module (van Kessel et al., 2025). Pathway-level BMCs were calculated by taking the median BMC of all qualifying genes within each gene set, provided that at least three genes were retained within the set, and 5% of the gene set is covered by those genes.

In addition to median-based aggregation of gene-level BMCs, pathway activity scores were modeled directly for the RPTEC/TERT1 WGCNA modules. Module eigengene scores were used as pathway-level response variables and modeled using the same BMC fitting and post-model filtering criteria described for the gene-level analysis. This approach estimates a BMC directly from the concentration-response profile of each module eigengene.

### BME-WTT-DESEQ-RF-S0 workflow

This workflow follows an approach that has been implemented in previous studies (Thienpont et al., 2024, 2025). Initially, this workflow was developed to derive transcriptomic Points of Departures (tPODs) for two biomarkers of genotoxicity called GENOMARK and TGx-DDI. Given that Cisplatin is a known genotoxicant, we found it relevant to include these two biomarkers in our analysis. Thus, choices in our workflow have been influenced by the analysis of genotoxic biomarkers.

#### TempO-Seq Data Preprocessing

First, probe-to-gene mapping was performed using the Human Whole Transcriptome 1.2 manifest (realigned in 2023). The relevance filter, adapted from the R-ODAF pipeline (Verheijen et al., 2022), was then applied, resulting in the removal of 8,477 probes. Probe counts mapping to the same gene were aggregated, yielding 11,531 unique genes for downstream analysis.

The resulting dataset was normalized using the DESeq2 R package. Normalized counts were log2-transformed for downstream analyses.

#### Concentration-Response Modeling

##### Gene Level Analysis

To conduct the BMC analysis, log2-normalized read counts were uploaded into BMDExpress3 (version 3.20), following the recommendations outlined in the U.S. NTP Approach to Genomic Dose-Response Modeling report (NTP, 2018; Phillips et al., 2019). Gene expression data were prefiltered using the Williams Trend Test, retaining genes with a permutation p-value < 0.01 (based on 500 permutations) and an absolute linear fold change (FC) > 1.5 for at least one concentration relative to matched controls. For genes passing these filters, concentration-response modeling was performed using following EPA BMDS models: Linear, Exponential (3 and 5), Polynomial (2nd degree), and restricted Power (exponent ≥ 1). In BMDExpress3, the optimal polynomial degree was determined using a nested chi-square test, after which the Akaike Information Criterion (AIC) was applied to select the best-fitting model, balancing goodness-of-fit with model complexity. The benchmark response (BMR) was set to 1 SD, following the recommendations of the U.S. NTP expert panel. BMC estimates were further filtered using quality criteria: only models with an R^2^ > 0.8, a BMC/BMCL ratio < 20, a BMCU/BMCL ratio < 40, and BMC values below the highest tested concentration (50 µM) were retained. Here, BMCL and BMCU represent the lower and upper confidence limits of the BMC, respectively.

##### Pathway Level Analysis

Gene set-level BMCs were derived for the Hallmark gene sets (2023 and the genotoxicity biomarker sets GENOMARK and TGx-DDI by calculating the median BMC of retained genes mapped to each gene set. For GENOMARK and TGx-DDI, median BMCs were calculated only when the biomarker model generated a positive prediction for at least one concentration at any time point. For Hallmark gene sets, median BMCs were calculated only for gene sets showing significant enrichment, defined as an adjusted p-vSalue < 0.05 for the normalized enrichment score (NES) in at least one concentration.

In parallel, Hallmark pathway activity scores were modeled directly by applying BMC modeling to the NES. In this score-based approach, NES values were treated as pathway-level response variables, and the same model-fitting and post-model filtering criteria described for the gene-level analysis were applied. This approach estimated a BMC directly from each Hallmark NES concentration-response profile rather than summarizing gene-level BMCs within the gene set.

### DRO-Quad-UQ-RF-S0 workflow

#### TempO-Seq Data Preprocessing

This workflow was developed for analyzing BioSpyder TempO-Seq count data, which has already undergone manufacturer platform-specific preprocessing steps (library preparation, base calling, raw data filtering, sequence alignment, gene quantification). Count data were preprocessed using R v4.3.3 (*limma* v3.56.2, *edgeR* v3.42.4, *dplyr* v1.1.4, and *baseR*) (Ritchie et al., 2015; Robinson et al., 2009). Quality control visualizations were used to assess the efficacy of preprocessing steps. Primarily, library sizes were calculated, and a threshold of 500,000 read counts was set. All samples passed library size cutoffs. Next, normalization methods were assessed for applicability and included no normalization (none), upper quartile (UQ), trimmed mean of M-values (TMM), TMM with singleton paring (TMMwsp), and relative log expression (RLE) [1]. After assessment, the UQ normalization method was applied (Li et al., 2020). A relevance filter was applied as per Verheijen *et al.* (Verheijen et al., 2022). Next, probes were mapped to the corresponding EntrezIDs and those corresponding to the same gene were summed. Correlation analysis showed improved correlation values after preprocessing and overall good accordance between controls.

#### Concentration-Response Modeling

##### Gene Level Analysis

BMC analysis was carried out using the *DRomics* v2.5-2 package in R to derive tPODs, using the quadratic trend test (Larras et al., 2018), with multiple testing correction using the Benjamini-Hochberg methods and a false discovery rate (FDR) threshold of 0.01 for concentration-responsive genes, and confidence interval calculations using 1,000 bootstrap iterations. A parametric modeling approach is performed by *DRomics*, whereby multiple models are fitted (linear, Hill, exponential, Gauss-probit, and log-Gauss-probit functions). This allows for the assessment of monotonicity and biphasic trends. The best fit model is selected based on the second order Akaike Information Criterion (AICc). Benchmark response was defined as 1 standard deviation (1 SD) from the control mean (zSD). A maximum of 1,000 iterations were allowed for model fitting, and a 95% confidence level with a tolerance of 0.5 was applied to derive BMC intervals. Items for which bootstrap confidence intervals could not be computed were excluded from downstream analysis.

#### Pathway Level Analysis

The R packages *KEGGrest* v1.40.1 and *clusterProfiler* v4.8.3 were used for pathway enrichment analysis using KEGG (2021) and MSigDB Hallmark (2021) databases (Kanehisa & Goto, 2000; Liberzon et al., 2015). Direct output from the BMC analysis generated gene-level BMC values. In addition, gene set/pathway-level BMC estimates were performed as per the U.S. NTP recommendations (National Toxicology Program 2018). For features to be included in gene set analysis, the following criteria were implemented for the best fit models: (1) must demonstrate convergent BMC, BMCL, and BMCU values; (2) the BMC must be less than the highest positive concentration used in the study; (3) it should not map to more than one gene; (4) the BMCU-to-BMCL ratio should be less than 40. Concentration-responsive items were filtered based on these criteria, and genes were parsed into sets that met the following conditions: contain at least 3 genes and populate at least 5% of the total annotated gene number.

### DRO-Quad-VST-RF-S0 workflow

#### TempO-Seq Data Preprocessing

R version 4.4.2 was used as the general platform for analyses. First, a quality control of the samples was done based on sequencing depth. A histogram of sequencing depths was made. No clear outliers were observed, and all samples had a sequencing depth larger than 500,000, so no samples were removed. Then, the data and metadata were loaded in DESeq2 (version 1.44.0). A factor with every unique combination of concentration and time was used to set up the design matrix. Afterwards, a relevance filter was applied to remove genes that had low expression and were not homogenously detected within their condition/group. More specifically, all genes that did not have at least 75% of their replicates expressed at 1 CPM in any one of the experimental conditions were excluded. A correlation matrix was made based on variance stabilized transformation (vst) result of the read counts. The replicate correlation was always higher than 80%, so no samples were removed. Finally, raw counts whose probes were mapped to the same entrez id were summed.

#### Concentration-Response Modeling

##### Gene Level Analysis

Concentration–response characterization was performed using **DRomics** (version 2.6.2) (Larras et al., 2018). RNA-seq count data were imported into DRomics with the function *RNAseqdata*(). As recommended, raw counts were used, and variance stabilization was applied with the *vst* option (variance-stabilizing transformation), which is preferred over *rlog* when the number of samples exceeds 30. This transformation automatically normalizes for library size and applies a log2 scale. Significantly responding genes were identified with the function *itemselect*(), using a quadratic trend test with a false discovery rate (FDR) threshold of 0.01. Concentration–response modeling was then carried out by fitting five families of models designed to capture monotonic and biphasic responses: Hill, linear, exponential, Gauss-probit, and log-Gauss-probit. For each gene, the best-fitting model was selected based on the second-order Akaike Information Criterion (AICc). Benchmark concentration (BMC) values were estimated using the BMD-zSD approach implemented in the function *bmdcalc()*, with z = 1. Confidence intervals for BMCs were obtained by bootstrapping with 1,000 iterations. Only genes with finite BMC point estimates and bounded confidence intervals were retained for downstream analyses.

#### Pathway Level Analysis

The hallmark gene sets form the molecular signature database were loaded in r with the function msigdbr(species = “Homo sapiens”, category = “H”) from the package “msigdbr” (version 7.5.1). The median and IQR for each of the gene sets were calculated, and only gene sets with at least 3 genes were retained.

### DRO-Quad-VST-C10-S0 workflow

#### TempO-Seq Data Preprocessing

All analyses were performed in R (version 4.4.2). Probe-to-gene mapping was conducted using the Human Whole Transcriptome 1.2 manifest (realigned in 2023). When multiple probes mapped to the same Entrez Gene ID, their counts were summed to obtain gene-level expression values. Prior to concentration–response analysis, a minimal expression filter was applied. Genes were retained if they had at least 10 raw counts in a minimum of three samples. No additional CPM-based relevance filtering was applied for the BMC modelling step.

Sequencing depth and sample relationships were assessed descriptively. All samples passed quality control and were retained for downstream analyses. Variance-stabilizing transformation (VST) from DESeq2 was used for exploratory analyses (e.g. PCA), but not for concentration–response modelling.

#### Concentration-Response Modeling

##### Gene Level Analysis

Concentration–response analysis was carried out using the DRomics package (version 2.5-2) in R (Larras et al., 2018). Raw gene-level counts were imported into DRomics by implementing the *formatdata4DRomics()* and *RNAseqdata()* functions. Internal normalization and transformation were performed within DRomics prior to modelling. Concentration-responsive genes were identified using the quadratic trend test, applying a Benjamini–Hochberg false discovery rate (FDR) threshold of 0.01. Parametric concentration–response models were fitted using the linear, Hill, exponential, Gauss–probit and log-Gauss–probit model families. For each gene, the best-fitting model was selected based on the second-order Akaike Information Criterion (AICc). Benchmark concentration (BMC) values were derived using the BMR-zSD approach, defining the benchmark response as a 1 standard deviation change from the control mean. Confidence intervals were estimated by bootstrap with 1000 iterations. Only genes with finite BMC confidence intervals were retained for subsequent analyses. No additional filtering based on BMC magnitude or BMC confidence interval width was applied.

##### Pathway Level Analysis

Over-representation analysis was performed separately for each timepoint using the lists of concentration-responsive genes with finite BMC confidence intervals. Enrichment analysis was conducted using Reactome (ReactomePA), KEGG, MSigDB Hallmark gene sets (msigdbr), and WikiPathways (clusterProfiler). Default significance thresholds implemented in these tools were applied (Benjamini–Hochberg adjusted p-values). For pathway-level BMC estimation, gene-level BMC values were mapped to gene sets and the median BMC (together with the corresponding median BMCL and BMCU) was calculated for each biological set.

## **References**

Callegaro, G., Kunnen, S. J., Trairatphisan, P., Grosdidier, S., Niemeijer, M., den Hollander, W., Guney, E., Piñero Gonzalez, J., Furlong, L., Webster, Y. W., et al. (2021). The human hepatocyte TXG-MAPr: gene co-expression network modules to support mechanism-based risk assessment. *Archives of Toxicology 2021 95:12*, *95*(12), 3745–3775. https://doi.org/10.1007/S00204-021-03141-W

Kanehisa, M., & Goto, S. (2000). KEGG: Kyoto Encyclopedia of Genes and Genomes. *Nucleic Acids Research*, *28*(1), 27. https://doi.org/10.1093/NAR/28.1.27

Langfelder, P., & Horvath, S. (2008). WGCNA: an R package for weighted correlation network analysis. *BMC Bioinformatics 2008 9:1*, *9*(1), 559-. https://doi.org/10.1186/1471-2105-9-559

Larras, F., Billoir, E., Baillard, V., Siberchicot, A., Scholz, S., Wubet, T., Tarkka, M., Schmitt-Jansen, M., Laure Delignette-Muller, M. (2018). DRomics: A Turnkey Tool to Support the Use of the Dose-Response Framework for Omics Data in Ecological Risk Assessment A Turnkey Tool to Support the Use of the Dose-Response Framework for Omics Data in Eco-logical Risk Assessment. *Environmental Science and Technology*, *52*(24), 14461–14468. https://doi.org/10.1021/acs.est.8b04752ï

Li, X., Cooper, N. G. F., O’Toole, T. E., & Rouchka, E. C. (2020). Choice of library size normalization and statistical methods for differential gene expression analysis in balanced two-group comparisons for RNA-seq studies. *BMC Genomics 2020 21:1*, *21*(1), 75-. https://doi.org/10.1186/s12864-020-6502-7

Liberzon, A., Birger, C., Thorvaldsdóttir, H., Ghandi, M., Mesirov, J. P., & Tamayo, P. (2015). The Molecular Signatures Database (MSigDB) hallmark gene set collection. *Cell Systems*, *1*(6), 417. https://doi.org/10.1016/J.CELS.2015.12.004

Love, M. I., Huber, W., & Anders, S. (2014). Moderated estimation of fold change and dispersion for RNA-seq data with DESeq2. *Genome Biology*, *15*(12), 550. https://doi.org/10.1186/S13059-014-0550-8

NTP. (2018). *NTP Research Report on National Toxicology Program Approach to Genomic Dose-Response Modeling*.

Phillips, J. R., Svoboda, D. L., Tandon, A., Patel, S., Sedykh, A., Mav, D., Kuo, B., Yauk, C. L., Yang, L., Thomas, R. S., et al. (2019). BMDExpress 2: enhanced transcriptomic dose-response analysis workflow. *Bioinformatics (Oxford, England)*, *35*(10), 1780–1782. https://doi.org/10.1093/BIOINFORMATICS/BTY878

Ritchie, M. E., Phipson, B., Wu, D., Hu, Y., Law, C. W., Shi, W., & Smyth, G. K. (2015). limma powers differential expression analyses for RNA-sequencing and microarray studies. *Nucleic Acids Research*, *43*(7), e47. https://doi.org/10.1093/nar/gkv007

Robinson, M. D., McCarthy, D. J., & Smyth, G. K. (2009). edgeR: a Bioconductor package for differential expression analysis of digital gene expression data. *Bioinformatics*, *26*(1), 139. https://doi.org/10.1093/bioinformatics/btp616

Thienpont, A., Cho, E., Williams, A., Meier, M. J., Yauk, C. L., Beal, M. A., Van Goethem, F., Rogiers, V., Vanhaecke, T., & Mertens, B. (2025). In vitro to in vivo extrapolation modeling to facilitate the integration of transcriptomics data into genotoxicity assessment. *Toxicology*, *515*. https://doi.org/10.1016/j.tox.2025.154165

Thienpont, A., Cho, E., Williams, A., Meier, M. J., Yauk, C. L., Rogiers, V., Vanhaecke, T., & Mertens, B. (2024). Unlocking the Power of Transcriptomic Biomarkers in Qualitative and Quantitative Genotoxicity Assessment of Chemicals. *Chemical Research in Toxicology*, *37*(3), 465–475. https://doi.org/10.1021/acs.chemrestox.3c00318

van Kessel, H. W., Kunnen, S. J., Callegaro, G., & van de Water, B. (2025). Advancing transcriptomics-based mechanistic assessment of nephrotoxicity in vitro using the human RPTEC/TERT1 TXG-MAPr gene co-expression network. *Toxicological Sciences*. https://doi.org/10.1093/TOXSCI/KFAF159

Verheijen, M. C., Meier, M. J., Asensio, J. O., Gant, T. W., Tong, W., Yauk, C. L., & Caiment, F. (2022). R-ODAF: Omics data analysis framework for regulatory application. *Regulatory Toxicology and Pharmacology*, *131*, 105143. https://doi.org/10.1016/J.YRTPH.2022.105143

Yang, L., Allen, B. C., & Thomas, R. S. (2007). BMDExpress: a software tool for the benchmark dose analyses of genomic data. *BMC Genomics 2007 8:1*, *8*(1), 387-. https://doi.org/10.1186/1471-2164-8-387

## **Supplemental Figures**

**Supplemental Figure 1. Cytotoxicity and concentration-range assessment of cisplatin exposure in RPTEC/TERT1 cells.** Cisplatin-induced Annexin V and propidium iodide (PI) positivity over time. Lines represent the tested cisplatin concentrations (**A**). Number of differentially expressed genes (DEGs) identified per cisplatin concentration and time point using the BMDE-noWTT-CPM-RF-S5 workflow. Bars indicate the number of significantly (FDR < 0.05 & |log2fc| >= log2(1.5) upregulated or downregulated genes, with bar direction and color denoting the direction of regulation.

**Alt text:** Cisplatin-induced cytotoxicity and transcriptional activity across concentrations and time points. Panel A shows Annexin V and PI positivity over time for different cisplatin concentrations. Panel B shows the number of upregulated and downregulated genes per concentration and time point.

**Supplemental Figure 2.** Comparison of genes excluded by prefiltering (Williams Trend Test, adjusted p-value < 0.05), and genes removed by post-model filtering (BMC >= 0.01, BMC <= 50, BMD/BMCL <= 20, BMCU/BMC <= 20, R-squared >= 0.8). Results are shown for the BMDE-noWTT-CPM-RF-S5 pipeline.

***Alt text:*** *Comparison of genes excluded by the Williams Trend Test prefiltering and genes removed during post-model filtering in the BMDE-noWTT-CPM-RF-S5 pipeline.*

**Supplemental figure 3.** Overlap of genes retained across analytical methods following prefiltering (left) and post-model filters (right). Colors represent different methods.

***Alt text:*** *Overlap of genes retained across analytical methods after prefiltering and post-model filtering.*

**Supplemental Figure 4. Permutation-based empirical null analysis of pre-model CRG identification.** Percentage of permuted datasets yielding at least one CRG, shown by workflow and time point. **(A)** Number of CRGs identified in each permuted dataset, shown by workflow and time point **(B)**.

***Alt text:*** *Supplemental figure showing the empirical null distribution of CRG counts after permutation of concentration labels. Panel A shows the percentage of permutations with at least one CRG for each workflow and time point. Panel B shows the number of CRGs identified in each permutation. Colors represent the different workflows.*

**Supplemental Figure 5.** Correlation plots of best-fit models within the BMDExpress workflows (BMDE-noWTT-CPM-RF-S5 & BME-WTT-DESEQ-RF-S0), illustrating model-specific patterns contributing to the accumulation of benchmark concentrations (BMCs).

***Alt text:*** *Correlation plots of best-fit models within the BMDExpress workflows, showing model-specific patterns that contribute to gene-level benchmark concentration (BMC) accumulation.*

**Supplemental Figure 6. Distribution of gene-level BMCs with corresponding** tPOD **estimates.** Vertical lines indicate the evaluated tPOD metrics (5^th^ percentile, 25^th^ ranked gene, first mode of the distribution, inflection point [Kneedle] and LCRD), with different line types and colors distinguishing the metrics. Rows represent the different timepoints.

***Alt text:*** *Distributions of gene-level benchmark concentrations (BMCs) with vertical lines marking tPOD estimates, including the 5^th^ percentile, 25^th^ ranked gene, first mode, inflection point (Kneedle), and LCRD.*

## **Supplemental Tables**

**Supplemental Table 1. Selected variables from the OECD Omics Reporting Framework (OORF).** This table presents a selection of key OORF variables used to compare the analytical workflows across partners. It serves as the main reference document for identifying methodological differences between workflows.

***Alt text:*** *Table summarizing selected variables from the OECD Omics Reporting Framework used to compare analytical workflows across partners. The table highlights key methodological differences between workflows.*

| Description | | DRO-Quad-UQ-RF-S0 | DRO-Quad-VST-RF-S0 | DRO-Quad-VST-C10-S0 | BMDE-noWTT-CPM-RF-S5 | BME-WTT-DESEQ-RF-S0 |
| --- | --- | --- | --- | --- | --- | --- |
| 3.2.3 Analysis of Raw Data | 3.2.3.3.i Raw Data filtering and trimming: Minimum read count | 500 000 | Histogram; no outliers | 500,000 | 500,000 (after probe QC), removed 1 sample | 500,000 |
|  | 3.2.3.5.iv Gene quantification: Description of method for summarizing transcript or probe counts to gene counts | Probes mapped to the same Entrez ID_id were summed | Probes mapped to the same Entrez ID were summed | Probes mapped to the same Entrez ID were summed | Probes mapped to the same Entrez ID were summed | Probes mapped to the same Entrez ID were summed |
| 3.2.4. Data Normalisation | 3.2.4.1.a Normalisation of the Raw Count method | Log2 Upper Quartile normalization | Log2 VST | Log2 VST | Log2 CPM | Log2 DESeq2 |
| 3.2.5. Post-Normalisation Data Filtering | 3.2.5.1.a&b Identification and Removal of Low Quality or Outlying Data Sets | PCA + correlation analysis to visualize, no samples removed | Replicate correlation < 0.8, No samples were removed | N/A | Replicate correlation - calculate pairwise correlations for all replicates in a treatment condition, find max correlation (X), remove samples if the max pairwise correlation of one replicate within the treatment condition is < X - 3*IQR. This removed 4 samples | Replicate correlation < 0.8: no samples were removed |
|  | 3.2.5.2.a Identification and Removal of Low Quality or Outlying Data Sets: Low read count filtering step | Relevance filter | Relevance filter | Minimum raw-count criterion (≥10 counts in at least 3 samples) | Relevance filter | Relevance filter |
| 4.1.5. Statistical Analysis to Identify Differentially Abundant Molecules | 4.1.5.1. Statistical Approach | N/A | NA | N/A | Deseq2 (+/-) | Deseq2 (+/-) |
| 4.3. Data Analysis Reporting Module (DARM) for Benchmark Dose Analysis and Quantification of Biological Potency. | 4.3.2.1. Software | DRomics (Version 2.5-2) | DRomics (Version 2.6-2) | DRomics (Version 2.5-2) | BMDExpress3 (Version: BMDExpress 3.20.0095 BETA) | BMDExpress3 (Version: BMDExpress 3.20.0095 BETA) |
|  | 4.3.4.1. Statistical Test Performed to Identify Dose-Responsive MSD | Quadratic | Quadratic | Quadratic | Williams Trend Test (but not used as pre-filter for BMD input) | Williams Trend test |
|  | 4.3.4.2. Statistical Threshold Applied | FDR < 0.01 | FDR < 0,01 | FDR < 0.01 | FDR < 0.05 (no exclusion critérium) | p-value < 0.01 & fold filter >= 1.5 |
|  | 4.3.4.3. Statistical Multiple Testing Correction Method | Benjamini-Hochberg | Benjamini-Hochberg | Benjamini-Hochberg | Benjamini-Hochberg | N/A |
|  | 4.3.4.4. Additional Statistical Test Parameters | N/A | N/A | N/A | Number of permutations: 10000 | Number of permutations: 500 |
|  | 4.3.4.5. Additional Filtering | N/A | N/A | N/A | N/A | N/A |
|  | 4.3.5.1. BMD Modeling Approach | Parametric | Parametric | Parametric | Parametric | Parametric |
|  | 4.3.5.2. Method for Final Estimation of MSD BMD | best model selection | best model selection | best model selection | best model selection | best model selection |
|  | 4.3.5.3. List of Models Fit to the Data | Linear, Hill, exponential, Gauss-probit and log-Gauss-probit (see Larras et al. 2018 for their definition) | Linear, Hill, exponential, Gauss-probit and log-Gauss-probit | linear, Hill, exponential, Gauss-probit and log-Gauss-probit | EPA BMDS models: hill, power, linear, poly 2, poly 3, exponential 3, exponential 5 | EPA BMDS models: power, linear, poly 2, exponential 3, exponential 5 |
|  | 4.3.5.4. Model Averaging Approach | N/A | N/A | N/A | *N/A* | N/A |
|  | 4.3.5.5. Method for Determining Benchmark Response (BMR; a.k.a. BMR Type) | BMR-zSD = y0 +/- z*SD, where y0 is the mean control response, and SD is the residual standard deviation of the considered CRC and z is the factor of SD | BMR-zSD = y0 +/- z*SD, where y0 is the mean control response, and SD is the residual standard deviation of the considered CRC and z is the factor of SD | BMR-zSD = y0 +/- z*SD, where y0 is the mean control response, and SD is the residual standard deviation of the considered CRC and z is the factor of SD | Standard Deviation | Standard Deviation |
|  | 4.3.5.6. Benchmark Response (BMR, a.k.a. BMR Factor) | z = 1, so 1SD | z = 1, so 1SD | z = 1, so 1SD | 1.021 (5%) | 1 SD |
|  | 4.3.5.7. Number of Fitting Iterations | Number of bootstrap to calculate confidence interval = 1000 | Number of bootstrap to calculate confidence interval = 1000 | Number of bootstrap to calculate confidence interval = 1000 | 250 | 250 |
|  | 4.3.5.8. Confidence Interval of the BMD (a.k.a. Confidence Level) | 0.95 with tolerance level of 0.5 | 0.95 | 0.95 | 0.95 | 0.95 |
|  | 4.3.5.9. Power Parameter Restriction | N/A | N/A | N/A | *>=1* | *>=1* |
|  | 4.3.5.10. Variance Assumption | *None* | *None* | None | Constant | Constant |
|  | 4.3.5.11. Model Selection | Best AICc | Best AICc | Best AICc | *Lowest AIC; models were only considered if (1) BMD/BMDL < 20, (2) BMDU/BMD < 20, (3) RSquared >= 0.8, (4) BMD between 1/10 of lowest dose and highest dose.* | Lowest AIC; models were only considered if (1) BMD <= Highest concentration, (2) BMD/BMDL < 20, (3) BMDU/BMDL < 40, (4) RSquared >= 0.8 |
|  | 4.3.5.12. Criteria to Identify Models that Extrapolate Outside the Dose Range | Finite BMD point estimates and CI bounds | Finite BMD point estimates and CI bounds | Finite BMD point estimates and CI bounds | Model was only considered when the Best BMD was between 1/10 of the lowest dose and highest concentration. | Model was only considered when BMD was lower than the highest concentration |
|  | 4.3.6.1. Biological Entity or Biological Set Annotation Used for the Analysis | Human KEGG (2021), MSigDB (Hallmark, 2021) | Hallmark gene sets from MSigDB (*msigdbr* package v25.1.1) | Hallmark (msigdbr package v25.1.1), WikiPathways (clusterProfiler 4.16.0) | RPTEC-TERT1 TXG-MAPr modules, MSigDB (Hallmark, 2023) | GENOMARK, TGxDDI biomarker sets, Hallmark (version 2023.2) |
|  | 4.3.6.2. MSD Acceptance Criteria for Use in Biological Entity of Set Analysis | (1) BMD <= 50; (2) BMDU/BMDL <= 40. | N/A | *N/A* | *N/A* | N/A |
|  | 4.3.6.3. Criteria for Identification of “Active” Biological Sets | Contain at least 3 genes and populate at least 5% of the total annotated gene number. | Gene sets that contained at least 3 genes were retained | Gene sets with an ORA padj < 0.1 were included | At least 3 genes passing all filters, >=5% coverage | GENOMARK and TGxDDI: positive predictions in at least one concentration of any time point.  HALLMARKS: NES associated padj < 0.05 |
|  | 4.3.6.4. Method of Estimating the BMD, BMDL, and BMDU of the Individual Biological Entity or Biological Sets | Median | Median | Median | Median | Median |

**Supplemental Table 2.** **Empirical null distribution of concentration-responsive gene (CRG) counts after permutation of concentration labels.** Summary of the permutation-based empirical null analysis performed at the pre-model concentration-responsive gene identification step. For each workflow and time point, concentration labels were permuted 100 times while preserving the original group sizes, and the corresponding workflow-specific CRG-identification criterion was reapplied. The table reports the observed CRG count in the original dataset, the percentage of permutations yielding at least one CRG, the median and range of CRG counts across the permuted datasets, and the empirical p-value.

***Alt text:*** *Table summarizing CRG counts from a permutation-based empirical null analysis. For each workflow and time point, the table compares the observed number of CRGs with the distribution of CRG counts from 100 permuted datasets, including the percentage of permutations with at least one CRG, the median and range of null CRG counts, and an empirical p-value.*

| Workflow | Time point | Observed CRGs | Permutations with ≥1 CRG (%) | Median null CRGs | Null CRG range | Empirical p-value |
| --- | --- | --- | --- | --- | --- | --- |
| BMDE-noWTT-CPM-RF-S5 | 4h | 196 | 1 | 0.0 | 0–572 | 0.0198 |
|  | 8h | 3,304 | 0 | 0.0 | 0–0 | 0.0099 |
|  | 16h | 3,532 | 1 | 0.0 | 0–277 | 0.0099 |
|  | 24h | 8,226 | 1 | 0.0 | 0–714 | 0.0099 |
|  | 48h | 7,273 | 0 | 0.0 | 0–0 | 0.0099 |
|  | 72h | 8,895 | 2 | 0.0 | 0–4876 | 0.0099 |
| BMDE-WTT-DESEQ-RF-S0 | 4h | 649 | 100 | 258.5 | 101–1850 | 0.1490 |
|  | 8h | 2,833 | 100 | 312.0 | 84–2896 | 0.0198 |
|  | 16h | 2,807 | 100 | 328.0 | 105–1930 | 0.0099 |
|  | 24h | 6,477 | 100 | 245.5 | 78–3293 | 0.0099 |
|  | 48h | 5,593 | 100 | 291.5 | 80–3254 | 0.0099 |
|  | 72h | 6,401 | 100 | 303.0 | 63–5330 | 0.0099 |
| DRO-Quad-UQ-RF-S0 | 4h | 70 | 0 | 0.0 | 0–0 | 0.0099 |
|  | 8h | 1,782 | 4 | 0.0 | 0–1 | 0.0099 |
|  | 16h | 2,183 | 1 | 0.0 | 0–1 | 0.0099 |
|  | 24h | 4,576 | 1 | 0.0 | 0–1 | 0.0099 |
|  | 48h | 4,743 | 0 | 0.0 | 0–0 | 0.0099 |
|  | 72h | 5,245 | 3 | 0.0 | 0–6 | 0.0099 |

| DRO-Quad-VST-C10-S0 | 4h | 3,618 | 10 | 0.0 | 0–223 | 0.0099 |
| --- | --- | --- | --- | --- | --- | --- |
|  | 8h | 5,123 | 19 | 0.0 | 0–430 | 0.0099 |
|  | 16h | 3,086 | 8 | 0.0 | 0–68 | 0.0099 |
|  | 24h | 5,801 | 7 | 0.0 | 0–498 | 0.0099 |
|  | 48h | 6,049 | 13 | 0.0 | 0–431 | 0.0099 |
|  | 72h | 6,562 | 20 | 0.0 | 0–802 | 0.0099 |
| DRO-Quad-VST-RF-S0 | 4h | 295 | 10 | 0.0 | 0–223 | 0.0099 |
|  | 8h | 2,347 | 19 | 0.0 | 0–430 | 0.0099 |
|  | 16h | 2,866 | 8 | 0.0 | 0–68 | 0.0099 |
|  | 24h | 5,298 | 7 | 0.0 | 0–498 | 0.0099 |
|  | 48h | 5,581 | 13 | 0.0 | 0–431 | 0.0099 |
|  | 72h | 6,028 | 20 | 0.0 | 0–802 | 0.0099 |

**Supplemental Table 3. Number and percentage of benchmark concentrations (BMCs) and distribution-based transcriptomic points of departure (tPODs) below the lowest tested concentration (0.1 µM).** Values are presented as the absolute number, followed in parentheses by the percentage of all obtained BMCs or tPODs for the corresponding analysis workflow.

***Alt text:*** *Table summarizing the number and percentage of gene-level benchmark concentrations and distribution-based transcriptomic points of departure estimated below the lowest tested cisplatin concentration of 0.1 µM. Values are presented by workflow and time point as counts with percentages in parentheses.*

|  |  | BMDE-noWTT-CPM-RF-S5 | BME-WTT-DESEQ-RF-S0 | DRO-Quad-UQ-RF-S0 | DRO-Quad-VST-C10-S0 | DRO-Quad-VST-RF-S0 |
| --- | --- | --- | --- | --- | --- | --- |
| **BMC < lowest tested concentration (0.1 µM)** | *4h* | *0 (0%)* | *0 (0%)* | *2 (3%)* | *9 (0%)* | *19 (7%)* |
|  | *8h* | 0 (0%) | 0 (0%) | 7 (0%) | 5 (0%) | 15 (1%) |
|  | *16h* | 1 (0%) | 0 (0%) | 5 (0%) | 13 (1%) | 9 (0%) |
|  | *24h* | 91 (3%) | 26 (1%) | 623 (15%) | 725 (16%) | 679 (15%) |
|  | *48h* | 6 (0%) | 4 (0%) | 89 (2%) | 100 (2%) | 101 (2%) |
|  | *72h* | 7 (0%) | 2 (0%) | 47 (1%) | 56 (1%) | 56 (1%) |
|  | | | | | | |
| **tPOD < lowest tested concentration (0.1 µM)** | *4h* | 0 (0%) | 0 (0%) | 0 (0%) | 1 (20%) | 2 (40%) |
|  | *8h* | 0 (0%) | 0 (0%) | 1 (20%) | 0 (0%) | 1 (20%) |
|  | *16h* | 0 (0%) | 0 (0%) | 0 (0%) | 1 (20%) | 1 (20%) |
|  | *24h* | 2 (40%) | 2 (40%) | 3 (60%) | 3 (60%) | 3 (60%) |
|  | *48h* | 1 (20%) | 1 (20%) | 2 (40%) | 2 (40%) | 2 (40%) |
|  | *72h* | 0 (0%) | 0 (0%) | 2 (40%) | 2 (40%) | 2 (40%) |

**Supplemental Table 4. Benchmark concentrations for the top 15 most sensitive Hallmark pathways for each workflow at 48 h.** Pathways are ranked by increasing BMC, highlighting the most sensitive pathway-level responses across workflows. The Hallmark p53 pathway is underlined.

***Alt text:*** *Table to compare benchmark concentrations for the 15 Hallmark pathways with the lowest BMCs per partner at 48 h.*

|  | BMDE-noWTT-CPM-RF-S5 | | BME-WTT-DESEQ-RF-S0 | | DRO-Quad-UQ-RF-S0 | | DRO-Quad-VST-C10-S0 | | DRO-Quad-VST-RF-S0 | |
| --- | --- | --- | --- | --- | --- | --- | --- | --- | --- | --- |
|  | **Hallmark pathway** | **BMC** | **Hallmark pathway** | **BMC** | **Hallmark pathway** | **BMC** | **Hallmark pathway** | **BMC** | **Hallmark pathway** | **BMC** |
| 1 | HALLMARK_P53_PATHWAY | 6.89 | HALLMARK_P53_PATHWAY | 9.21 | HALLMARK_E2F_TARGETS | 3.76 | HALLMARK_E2F_TARGETS | 2.36 | HALLMARK_E2F_TARGETS | 2.36 |
| 2 | HALLMARK_SPERMATOGENESIS | 8.38 | HALLMARK_TNFA_SIGNALING_VIA_NFKB | 9.42 | HALLMARK_G2M_CHECKPOINT | 8.32 | HALLMARK_G2M_CHECKPOINT | 4.17 | HALLMARK_G2M_CHECKPOINT | 4.79 |
| 3 | HALLMARK_APOPTOSIS | 9.20 | HALLMARK_ESTROGEN_RESPONSE_LATE | 9.53 | HALLMARK_TNFA_SIGNALING_VIA_NFKB | 9.65 | HALLMARK_HYPOXIA | 4.82 | HALLMARK_HYPOXIA | 6.81 |
| 4 | HALLMARK_TNFA_SIGNALING_VIA_NFKB | 9.22 | HALLMARK_APOPTOSIS | 9.77 | HALLMARK_EPITHELIAL_MESENCHYMAL_TRANSITION | 9.97 | HALLMARK_APICAL_JUNCTION | 6.77 | HALLMARK_MITOTIC_SPINDLE | 7.33 |
| 5 | HALLMARK_EPITHELIAL_MESENCHYMAL_TRANSITION | 9.35 | HALLMARK_EPITHELIAL_MESENCHYMAL_TRANSITION | 9.93 | HALLMARK_INFLAMMATORY_RESPONSE | 10.41 | HALLMARK_GLYCOLYSIS | 6.89 | HALLMARK_KRAS_SIGNALING_DN | 7.73 |
| 6 | HALLMARK_PANCREAS_BETA_CELLS | 9.56 | HALLMARK_PI3K_AKT_MTOR_SIGNALING | 9.97 | HALLMARK_HYPOXIA | 10.70 | HALLMARK_ANGIOGENESIS | 7.06 | HALLMARK_APICAL_JUNCTION | 7.74 |
| 7 | HALLMARK_PI3K_AKT_MTOR_SIGNALING | 9.56 | HALLMARK_PEROXISOME | 10.31 | HALLMARK_COAGULATION | 10.77 | HALLMARK_MITOTIC_SPINDLE | 7.31 | HALLMARK_GLYCOLYSIS | 7.88 |
| 8 | HALLMARK_INFLAMMATORY_RESPONSE | 9.68 | HALLMARK_PANCREAS_BETA_CELLS | 10.78 | HALLMARK_KRAS_SIGNALING_UP | 11.01 | HALLMARK_TNFA_SIGNALING_VIA_NFKB | 8.23 | HALLMARK_TNFA_SIGNALING_VIA_NFKB | 8.20 |
| 9 | HALLMARK_UV_RESPONSE_UP | 9.86 | HALLMARK_E2F_TARGETS | 11.45 | HALLMARK_P53_PATHWAY | 11.39 | HALLMARK_COAGULATION | 8.37 | HALLMARK_EPITHELIAL_MESENCHYMAL_TRANSITION | 8.63 |
| 10 | HALLMARK_E2F_TARGETS | 9.92 | HALLMARK_G2M_CHECKPOINT | 11.51 | HALLMARK_ESTROGEN_RESPONSE_LATE | 11.53 | HALLMARK_KRAS_SIGNALING_UP | 8.40 | HALLMARK_XENOBIOTIC_METABOLISM | 9.22 |
| 11 | HALLMARK_HYPOXIA | 10.64 | HALLMARK_XENOBIOTIC_METABOLISM | 11.68 | HALLMARK_OXIDATIVE_PHOSPHORYLATION | 11.72 | HALLMARK_EPITHELIAL_MESENCHYMAL_TRANSITION | 8.64 | HALLMARK_SPERMATOGENESIS | 9.39 |
| 12 | HALLMARK_G2M_CHECKPOINT | 10.74 | HALLMARK_OXIDATIVE_PHOSPHORYLATION | 11.82 | HALLMARK_HEDGEHOG_SIGNALING | 12.13 | HALLMARK_KRAS_SIGNALING_DN | 8.77 | HALLMARK_KRAS_SIGNALING_UP | 9.56 |
| 13 | HALLMARK_XENOBIOTIC_METABOLISM | 10.78 | HALLMARK_IL6_JAK_STAT3_SIGNALING | 11.89 | HALLMARK_MYC_TARGETS_V1 | 12.17 | HALLMARK_SPERMATOGENESIS | 9.07 | HALLMARK_ESTROGEN_RESPONSE_LATE | 9.59 |
| 14 | HALLMARK_DNA_REPAIR | 10.83 | HALLMARK_MYOGENESIS | 12.40 | HALLMARK_ANDROGEN_RESPONSE | 12.53 | HALLMARK_XENOBIOTIC_METABOLISM | 9.08 | HALLMARK_OXIDATIVE_PHOSPHORYLATION | 9.86 |
| 15 | HALLMARK_ESTROGEN_RESPONSE_LATE | 11.05 | HALLMARK_BILE_ACID_METABOLISM | 12.46 | HALLMARK_APOPTOSIS | 12.53 | HALLMARK_P53_PATHWAY | 9.67 | HALLMARK_P53_PATHWAY | 9.96 |
